# The Origins and Vulnerabilities of Two Transmissible Cancers in Tasmanian Devils

**DOI:** 10.1016/j.ccell.2018.03.013

**Published:** 2018-04-09

**Authors:** Maximilian R. Stammnitz, Tim H.H. Coorens, Kevin C. Gori, Dane Hayes, Beiyuan Fu, Jinhong Wang, Daniel E. Martin-Herranz, Ludmil B. Alexandrov, Adrian Baez-Ortega, Syd Barthorpe, Alexandra Beck, Francesca Giordano, Graeme W. Knowles, Young Mi Kwon, George Hall, Stacey Price, Ruth J. Pye, Jose M.C. Tubio, Hannah V.T. Siddle, Sukhwinder Singh Sohal, Gregory M. Woods, Ultan McDermott, Fengtang Yang, Mathew J. Garnett, Zemin Ning, Elizabeth P. Murchison

**Affiliations:** 1 Transmissible Cancer Group, Department of Veterinary Medicine, University of Cambridge, Cambridge CB3 0ES, UK; 2 Mount Pleasant Laboratories, Tasmanian Department of Primary Industries, Parks, Water and the Environment, Prospect, TAS 7250, Australia; 3 School of Health Sciences, Faculty of Health, University of Tasmania, Launceston, TAS 7248, Australia; 4 Wellcome Trust Sanger Institute, Wellcome Genome Campus, Hinxton CB10 1SA, UK; 5 Menzies Institute for Medical Research, University of Tasmania, Hobart, TAS 7000, Australia; 6 Centre for Biological Sciences, University of Southampton, Southampton SO17 1BJ, UK

**Keywords:** cancer, Tasmanian devils, transmissible cancer, contagious cancer, DFTD, cancer evolution, cancer genomics, drug screening, conservation, marsupials

## Abstract

Transmissible cancers are clonal lineages that spread through populations via contagious cancer cells. Although rare in nature, two facial tumor clones affect Tasmanian devils. Here we perform comparative genetic and functional characterization of these lineages. The two cancers have similar patterns of mutation and show no evidence of exposure to exogenous mutagens or viruses. Genes encoding PDGF receptors have copy number gains and are present on extrachromosomal double minutes. Drug screening indicates causative roles for receptor tyrosine kinases and sensitivity to inhibitors of DNA repair. Y chromosome loss from a male clone infecting a female host suggests immunoediting. These results imply that Tasmanian devils may have inherent susceptibility to transmissible cancers and present a suite of therapeutic compounds for use in conservation.

## Highlights

• Tasmanian devil transmissible cancers arose from similar tissues in two individuals
• Similar mutation patterns and driver candidates imply common oncogenic processes
• Losses at *B2M* and Y chromosome loci suggest selection to escape immune detection
• Receptor tyrosine kinases and DNA repair factors implicated as therapeutic targets

## Graphical Abstract

**Figure:**
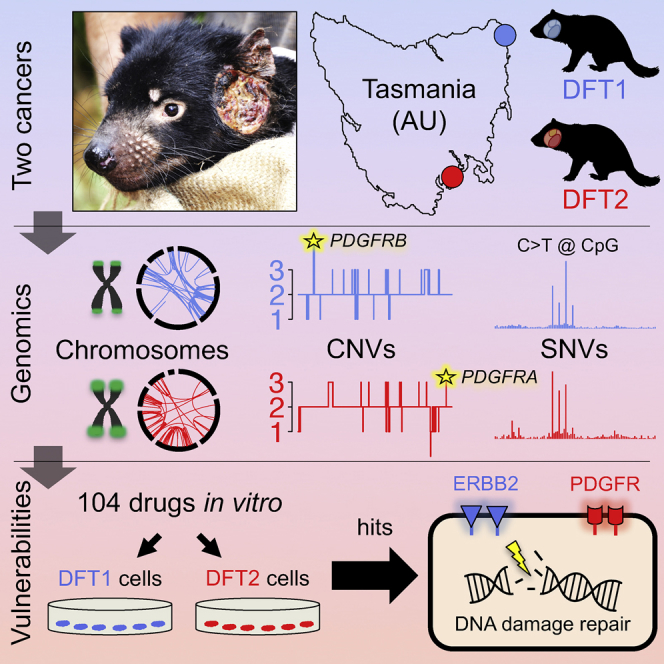

## Teaser

Stammnitz et al. show that the two transmissible cancer clones that affect Tasmanian devils are very similar in their tissues-of-origin, mutational patterns and driver gene candidates. Importantly, these cancers are both highly sensitive to inhibitors of some receptor tyrosine kinases as well as to inhibitors of DNA repair.

## Significance

**Transmissible cancers are malignant clones that “metastasize” between individuals. The mechanisms whereby such cancers emerge, spread, and escape the allogeneic immune system are poorly understood. Remarkably, despite the rarity of known transmissible cancers in nature, Tasmanian devils harbor two distinct transmissible facial tumor clones. Here, we investigate the underlying mechanisms of devil transmissible cancers by performing comparative genetic and functional analyses of the two clones. The cancers show striking similarities in their tissues-of-origin, genome architectures, mutational patterns, driver gene candidates, and drug vulnerabilities, suggesting that they arose via similar processes. Both cancers carry deletions at loci relevant for immunogenicity. Importantly, common dependence on receptor tyrosine kinases and DNA repair pathways provides opportunities for targeted therapy and Tasmanian devil conservation.**

## Introduction

Tasmanian devils (*Sarcophilus harrisii*) are marsupial carnivores endemic to the Australian island of Tasmania. This species is considered endangered due to the emergence of a clonally transmissible cancer known as devil facial tumor 1 (DFT1) (Pearse and Swift, 2006). DFT1 presents as facial and oral tumors, and the disease is contagious between animals by the transfer of living cancer cells by biting (Hamede et al., 2013, Pearse and Swift, 2006). First observed in north-east Tasmania in 1996, DFT1 is a somatic clone that originally arose from the cells of an individual female devil (Deakin et al., 2012, Hawkins et al., 2006, Murchison et al., 2012). The lineage spawned by this animal has subsequently spread widely throughout Tasmania, causing significant declines in devil populations (Hawkins et al., 2006, Lazenby et al., 2018).

In 2014, routine diagnostic screening revealed a second transmissible cancer in Tasmanian devils (Pye et al., 2016b). This cancer, DFT2, causes oral and facial tumors that are grossly indistinguishable from those caused by DFT1 (Pye et al., 2016b). However, DFT2 tumors are histologically, cytogenetically, and genetically distinct from DFT1. Indeed, karyotype evidence suggests that DFT2 arose from the somatic cells of a male animal, in contrast to the female origin of DFT1 (Pye et al., 2016b). To date, DFT2 has been confirmed in only 11 devils, all located on the Channel Peninsula in Tasmania's south-east (Kwon et al., 2018).

The discovery of a second transmissible cancer in Tasmanian devils was entirely unexpected and remains unexplained. Other than DFT1 and DFT2 in devils, only one other naturally occurring transmissible cancer is known in mammals, which is the 11,000-year-old canine transmissible venereal tumor in dogs (Murchison et al., 2014). Outside of mammals, only five transmissible cancers have been observed, all of which cause leukemia-like diseases in marine bivalves (Metzger et al., 2015, Metzger et al., 2016). The scarcity of known transmissible cancers in nature suggests that such diseases emerge rarely. Furthermore, in Tasmanian devils, there were no reports of animals with facial tumors comparable with those caused by DFT1 and DFT2 prior to 1996 (Hawkins et al., 2006, Loh et al., 2006a). Thus, the recent identification of two transmissible cancers in Tasmanian devils, detected within an interval of 18 years, is very surprising, and suggests that exogenous or anthropogenic factors may contribute to risk of transmissible cancer development specifically in this species.

Despite an urgent need to further understand the molecular basis of transmissible cancers in Tasmanian devils, little is known of the underlying genetic changes that initially caused these cancers and that promote their colonization of allogeneic hosts. The genome of DFT1 indicates that this lineage has acquired several thousand mutations during its evolution (Murchison et al., 2012). Although some genes have been somatically altered (Miller et al., 2011, Murchison et al., 2012, Taylor et al., 2017), no “driver” mutations with a clear causative role in DFT1 emergence or evolution have been identified. Major histocompatibility complex (MHC) molecules are undetectable on the surface of most DFT1 cells, likely explaining the low immunogenicity of these cells in allogeneic hosts (Siddle et al., 2013). However, no mutations in genes involved in antigen presentation have been defined. DFT2 has not yet been characterized beyond a preliminary assessment of its histology, karyotype, and genetic profiles at microsatellite and MHC loci (Pye et al., 2016b).

Given the similar phenotypes of DFT1 and DFT2, the emergence of DFT2 provides an opportunity to understand the common factors that underlie transmissible cancers in Tasmanian devils. Here, we provide a comparative genetic and functional characterization of DFT1 and DFT2, analyzed alongside 46 normal devil genomes.

## Results

### Tissues-of-Origin

DFT2 tumors are histologically distinct from those of DFT1 (Pye et al., 2016b). DFT2 is characterized by sheets of pleomorphic cells (amorphic to stellate and fusiform), whereas DFT1 is composed of pleomorphic round cells often arranged in bundles, cords, or packets (Loh et al., 2006a, Pye et al., 2016b). DFT1 expresses neuroectodermal markers, and is proposed to be of Schwann cell origin; indeed, a Schwann cell marker, PRX, is used to confirm DFT1 diagnosis (Loh et al., 2006b, Murchison et al., 2010, Tovar et al., 2011). DFT2 does not express PRX (Pye et al., 2016b) and its histogenesis remains unknown.

We used a panel of antibodies to broadly characterize the DFT2 tissue-of-origin by immunohistochemistry. Similar to DFT1, DFT2 is negative for cytokeratin and smooth muscle actin, and positive for vimentin, neural-specific enolase, and S100 (Figure S1). The similarity in tissue markers and gross phenotype between DFT1 and DFT2 suggests that these cancers arose from a similar cell type.

### Germline Genotypes and Populations-of-Origin

To further understand the identities, locations and relationship between the DFT1 and DFT2 founder individuals, whose cells spawned the two lineages, we compared the germline alleles present in DFT1 and DFT2 with those in the devil population.

**Figure 1:**
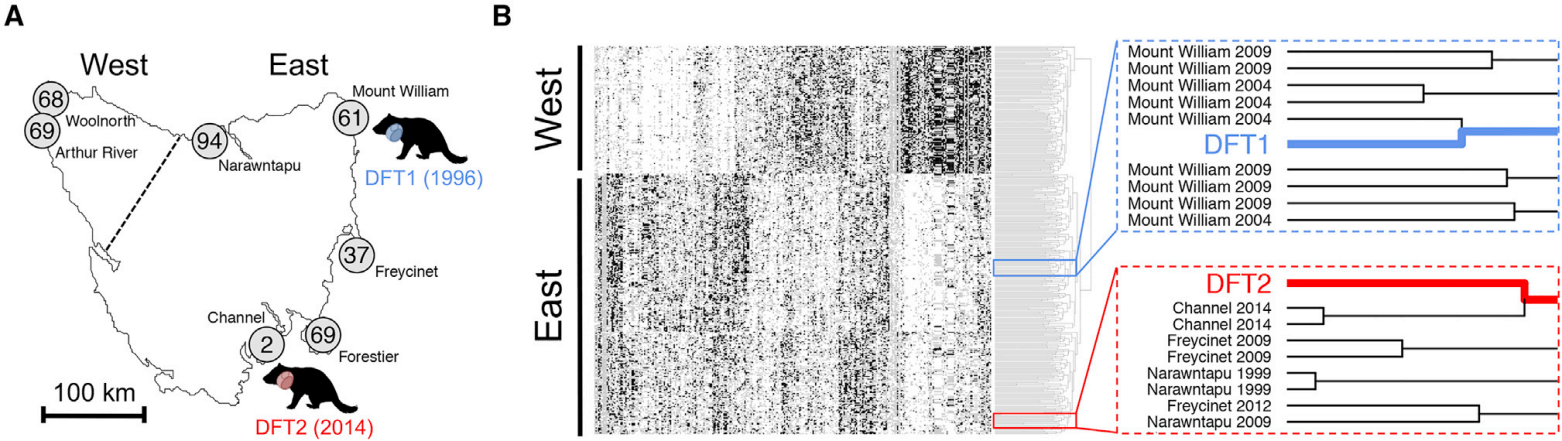
Origins of DFT1 and DFT2 (A) Map of Tasmania illustrating sampling locations of 400 devil individuals represented in (B). Number of individuals sampled from each location is labeled (Brüniche-Olsen et al., 2016). “East” and “West” denote the populations that cluster separately in (B), separated by dotted line. One individual was sampled from a captive population and is not shown on map. Devil silhouettes depict locations and year of first observations of DFT1 and DFT2. (B) Hierarchical clustering of 320 SNP genotypes across a panel of 401 devils, DFT1 (blue) and DFT2 (red); individuals are represented as rows and loci as columns. Genotypes are coded as white (homozygous 1/1), black (homozygous 2/2), and gray (heterozygous 1/2). East and West populations, as defined in (A), are labeled. Right, detail of Euclidian distance dendrogram with sampling years and locations of devils neighboring DFT1 and DFT2 genotypes. See also Table S1 and Figure S1.

Tasmanian devil genetic analysis has revealed population substructure between eastern devil populations and those in the more isolated north-west (Brüniche-Olsen et al., 2014, Jones et al., 2004, Miller et al., 2011). Genotyping of DFT1 and DFT2 (Table S1) at 320 nuclear polymorphic loci, and comparison with 401 devils sampled from seven locations between 1999 and 2014 (Brüniche-Olsen et al., 2016), confirmed that both DFT1 and DFT2 arose from individuals with “eastern” genotypes (Figure 1). Further analysis indicated that DFT1 clustered most strongly with individuals sampled in north-east Tasmania (Mount William) in 2004, whereas DFT2 bore closest identity with individuals collected in 2014 from the Channel Peninsula (Figure 1). Overall, these findings are consistent with the notion that DFT1 and DFT2 arose within the areas in which they were first observed (Figure 1B), implying that both lineages may have been discovered relatively soon after their emergence.

The independent emergence of transmissible cancers from two Tasmanian devils both belonging to the eastern subpopulation suggests the possibility of inherited germline predisposition alleles that increase risk of transmissible cancer development. We investigated this hypothesis by sequencing the genomes of DFT1 and DFT2 (Table S1) and identifying and annotating their founder individuals' inherited germline single-nucleotide variant (SNV) and small insertion and deletion (indel) alleles (variants were considered likely to be germline if they were shared with ≥1 normal devil or if they were found in both DFT1 and DFT2, see the STAR Methods). Although a subset of these caused putative non-synonymous gene alterations in 908 genes (Table S1), none bore homology to known inherited cancer risk loci in humans (Forbes et al., 2015). Overall, although this approach revealed a number of candidate loci, we cannot confirm their involvement in DFT risk.

### Virus Screen

We next investigated the possibility that exposure to exogenous pathogens, such as viruses, may increase the risk of DFT diseases developing in Tasmanian devils. We produced *de novo* assemblies of two DFT1 and two DFT2 genomes, and used whole genome and short read alignments to identify contigs that were exclusive to tumors and absent from four normal devils (see STAR Methods). This approach did not provide evidence for exogenous viral DNA in DFT1 or DFT2 (Table S1), consistent with the results of previous screens for viruses in DFT1 using sequence alignments and transmission electron microscopy (Murchison et al., 2012, Pyecroft et al., 2007). However, we cannot exclude the potential involvement of DNA viruses that have not been maintained, small circular unintegrated DNA viruses not captured by our DNA extraction method, RNA viruses, or other pathogens in triggering DFT emergence.

### Mutational Signatures

**Figure 2:**
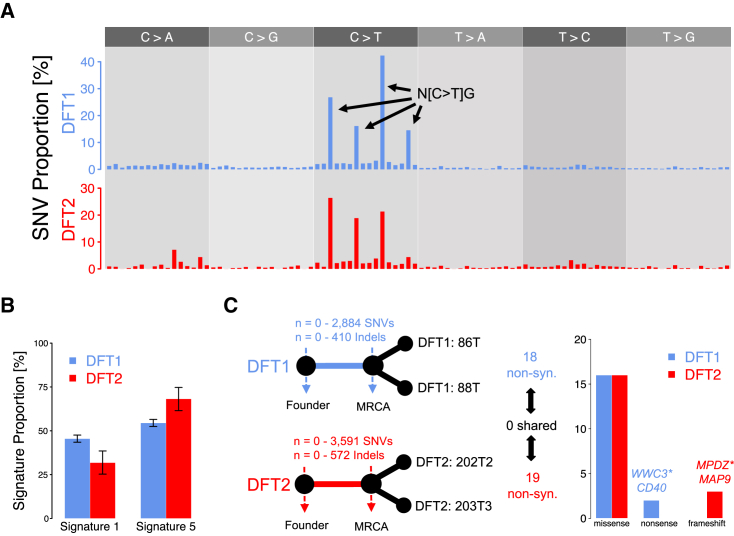
Single-Nucleotide Variants and Indels in DFT1 and DFT2 (A) Mutational spectra of single-nucleotide variants (SNVs). Only SNVs that are unique to one tumor within a lineage, and therefore likely to be somatic, are displayed (n = 6,812 [DFT1], n = 626 [DFT2]). Each bar represents a mutation category defined by the mutation type shown in upper gray panel, and its immediate 5′ and 3′ base context; mutation classes are presented in the order shown in (COSMIC, 2017), and prominent mutation types are labeled (N, any base). Mutation counts are normalized to corresponding nucleotide triplet frequencies in the devil genome. (B) Best fit of two mutational signatures. Signatures 1 and 5, extracted from human cancers (Alexandrov et al., 2013), were fitted to SNVs derived from DFT1 and DFT2. DFT1 and DFT2 SNVs were represented by a pool of those that are unique to one tumor within each lineage. Error bars display 95% Bayesian credible intervals of the posterior probability after 10^5^ Markov Chain Monte Carlo samples. (C) Analysis of early somatic variants. Left, simplified phylogenetic trees represent origins of DFT1 and DFT2 from their respective founder devils, and their respective divergence after the most recent common ancestor (MRCA) of the tumor isolates analyzed here (86T and 88T, DFT1) and (202T2 and 203T3, DFT2). Plausible range of somatic SNV and indel counts within the trunk of each tree is indicated, with the upper bound defined by those variants shared between both tumor isolates in each lineage but not detected in 46 normal devil genomes. The upper bounds of early somatic non-synonymous mutations in each lineage is shown and, right, annotation of these variants is represented. ^∗^ indicates the truncating mutations in *WWC3* and *MPDZ* are hemizygous as in both cases the second allele has been deleted. See also Tables S2, S3, and S4.

Further evidence for the involvement of exogenous agents in DFT1 and DFT2 pathogenesis might be gained from examination of mutational signatures (Alexandrov et al., 2013, Alexandrov et al., 2015a, Baez-Ortega and Gori, 2017). The similarity in mutational spectra, a representation of the six SNV mutation types together with their immediate 5′ and 3′ contexts found in DFT1 and DFT2 tumors, suggests that similar mutational processes have operated in these two cancers (Figure 2A). We applied Markov Chain Monte Carlo sampling with a Bayesian statistical model to refit the 30 mutational signatures cataloged in human cancers (COSMIC, 2017) to pools of mutations in DFT1 and DFT2. This analysis revealed that refitting with human mutational signatures 1 and 5, both of which are “clock-like” age-associated signatures, which are almost universally active in human cancer and normal cells and are not indicative of exogenous mutational exposures (Alexandrov et al., 2013, Alexandrov et al., 2015a, Blokzijl et al., 2016, Ju et al., 2017, Rahbari et al., 2016), adequately reconstructed the mutational spectra observed in both DFT1 and DFT2 (cosine similarity 0.93 and 0.95, respectively) (Figure 2B; Table S2).

Interestingly, neither DFT1 nor DFT2 genomes analyzed here bear imprints of exposure to UV light, a mutagen that leaves a readily recognizable mutational signature (Table S2). This contrasts with the transmissible venereal tumor in dogs, in which ∼40% of mutations have been caused by UV (Murchison et al., 2014). Given that both DFT1 and DFT2 tumors are frequently located on external regions of the face, this observation suggests that either the nocturnal Tasmanian devil is rarely exposed to UV or, alternatively, that the cells that propagate DFT1 and DFT2 are not those on the surface of cutaneous tumors, but rather derive from non-exposed regions, such as the oral cavity or deep within the tumor mass.

### Early Somatic Mutations

Our analysis has not provided evidence that exogenous exposures or germline risk contributed to DFT emergence. Next, we further characterized the functional consequences of putative somatic mutations in the two cancers. We identified 2,884 SNVs and 410 indels (DFT1), and 3,591 SNVs and 572 indels (DFT2), which were present in the genomes of two sequenced DFT1 tumors (86T and 88T, collected from Central Tasmania in 2005 and Eastern Tasmania in 2007, respectively) or two sequenced DFT2 tumors (202T2 and 203T3, both collected from the Channel Peninsula in 2014), but were not detected in the genomes of 46 normal devils (Figure 2C; Table S2). As we do not have germline DNA from the DFT1 or DFT2 founder devils, we cannot ascertain the provenance of these variants; however, a subset will be early somatic variants that occurred after emergence of each lineage and prior to divergence of the tumor isolates analyzed here (Figure 2C). Only 18 (18 SNVs, 0 indels) of these variants in DFT1 and 19 (16 SNVs, 3 indels) in DFT2 were predicted to be non-synonymous, with no intersection between the genes harboring non-synonymous variants in DFT1 and DFT2 (Figure 2C; Table S2). None of these putative early somatic non-synonymous SNV or indel mutations occurred in a set of genes with confirmed causative involvement in human cancer (http://cancer.sanger.ac.uk/cosmic/census/) (Tables S2 and S3). The majority of these mutations were predicted to be heterozygous (Table S2). However, we observed that DFT1 harbored a hemizygous nonsense mutation in *WWC3* (R945^∗^ in exon 21/24), and DFT2 carried a hemizygous truncating indel in *MPDZ* (S496X in exon 9/47); in both cases, the second copy was deleted, likely leading to complete loss-of-function (Tables S2 and S4). We genotyped these variants across eight additional geographically dispersed DFT1 tumors (*WWC3*) and two additional DFT2 tumors (*MPDZ*) (Table S2); in both cases, the relevant variant was present in all tumors analyzed, suggesting that these variants may be somatic mutations acquired early, prior to clonal diversification. Interestingly, both *WWC3* and *MPDZ* are proposed to encode negative regulators of YAP1 and WWTR1/TAZ, core effectors of the Hippo signaling pathway, which has conserved roles in development, regeneration, and cancer (Han et al., 2017, Juan and Hong, 2016, Moroishi et al., 2015, Varelas et al., 2010, Zanconato et al., 2016). YAP1 and WWTR1/TAZ are transcriptional co-activators that shuttle between cytoplasm and nucleus; in both DFT1 and DFT2 cells, YAP1 and WWTR1/TAZ are expressed and show nuclear localization, indicating activity (Figure S1). The Hippo pathway has been implicated in several human cancer histotypes, and is of particular importance in Schwann cell cancers (Wu et al., 2018, Zanconato et al., 2016).

### Cytogenetics and Structural Variants

**Figure 3:**
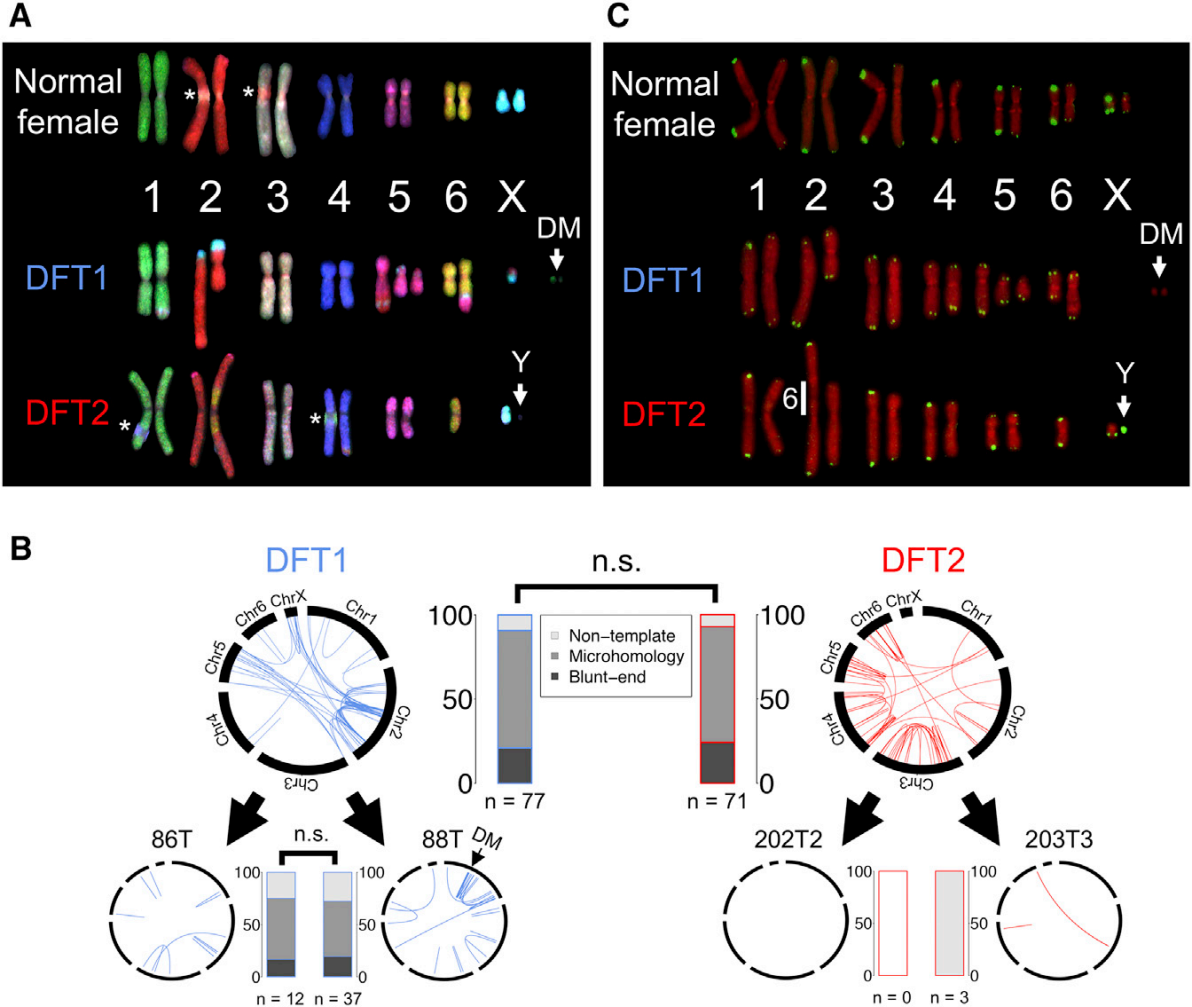
Structural Variation and Telomeres in DFT1 and DFT2 (A) Chromosome painting. Normal devil female, DFT1 (tumor 88T) and DFT2 (tumor 203T3) metaphases hybridized with devil chromosome-specific fluorescent probes. DM, double minutes; the Y chromosome lacks a specific probe and is indicated with “Y”; ^∗^ indicates locations of overlap between chromosome arms that were present in images used to generate karyotypes. (B) Structural variant (SV) mutations. Larger upper circos plots represent likely somatic SVs shared between 86T and 88T (DFT1) or 202T2 and 203T3 (DFT2), respectively, but that are not found in 34 normal devils. Lower circos plots represent SVs that are uniquely found in one of the sequenced tumors of the two lineages. DM, SVs involved in double minutes. Blue or red lines connect chromosomal coordinates involved in SV. Stacked bar plots indicate percentage of breakpoints displaying short regions of microhomology, non-templated sequence insertions or blunt ends. n.s., Pearson's chi square test, p > 0.05. (C) Telomeres. Normal devil female, DFT1 (tumor 88T) and DFT2 (tumor 202T2) metaphases hybridized with telomere-specific fluorescent probes (green). Chromosomes are labeled red. DMs and Y chromosome are indicated, as well as site of integration of chromosome 6 into the derivative chromosome 2 in DFT2. See also Tables S3 and S5.

Structural variants (SVs) are another source of somatic variation that may have contributed to DFT oncogenesis. Chromosome painting revealed that the DFT2 karyotype (Pye et al., 2016b) appears to have arisen via insertion of chromosome 6 into the pericentric region of chromosome 2, forming a large derived chromosome (Figure 3A). We used discordantly mapped paired-end sequence reads and PCR screens to identify putative somatic SVs in DFT1 and DFT2. The pattern of SVs in DFT1 revealed a cluster of rearrangements on chromosome 2 that was acquired prior to divergence of the tumors sequenced in this study (Figure 3B; Table S5). We also identified a focus of SVs on chromosome 1 in one DFT1 tumor, which marks the region from which the extrachromosomal double minutes (DMs) in this tumor derive (Taylor et al., 2017) (Figures 3A and 3B; Table S5). We identified 64 and 23 rearrangements involving genes in one or both DFT1 genomes or in one or both DFT2 genomes analyzed here, respectively, but not in 34 normal devil genomes (Table S5). These predicted three DFT1-specific in-frame fusion genes, *PDZD11*-*RFX2*, *CAMK2A*-*NEURL1B*, and *EZH2*-*ETNK2;* the latter two potential fusion genes were found in only one of two analyzed DFT1 tumors, and are thus unlikely to have arisen early in DFT1 tumor evolution (Tables S3 and S5). Genotyping of *PDZD11*-*RFX2*, however, confirmed its presence in eight additional geographically dispersed DFT1 tumors (Table S2), suggesting that it may be a somatic rearrangement that occurred early in the DFT1 lineage. *EZH2*, encoding a histone methyltransferase, is dysregulated in many cancers (Kim and Roberts, 2016), but it is unclear if the disruption of this gene in a subset of DFT1s has provided a selective advantage to this lineage (Table S3). Overall, the DFT2 genomes analyzed here have simpler structures and fewer rearrangements than those of the DFT1 genomes analyzed here. However, similar microhomology-mediated repair processes operated during clonal evolution of both DFT1 and DFT2 (Figure 3B; Table S5).

### Telomeres

Rearrangements in cancer are frequently triggered by telomere crisis (Maciejowski and de Lange, 2017). Tasmanian devils have unusual telomeres characterized by extreme length dimorphism between homologs (Bender et al., 2012). This feature has been lost in DFT1, which carries uniformly short telomeres (Bender et al., 2012). We used fluorescence *in situ* hybridization (FISH) to examine telomere length in DFT2. Our analysis revealed that cells derived from DFT2 exhibited telomere length dimorphism between homologs similar to normal cells (Figure 3C), and indicated that it was the chromosome 6 homolog with short telomeres that was incorporated into chromosome 2 to generate the large derivative chromosome in DFT2 (Figure 3C). Thus, although loss of telomere length dimorphism is not essential for the emergence of transmissible cancers in Tasmanian devils, this species' unusual telomere organization may contribute to risk of chromosomal rearrangement, which may predispose to DFT cancer.

### Copy Number Variants

**Figure 4:**
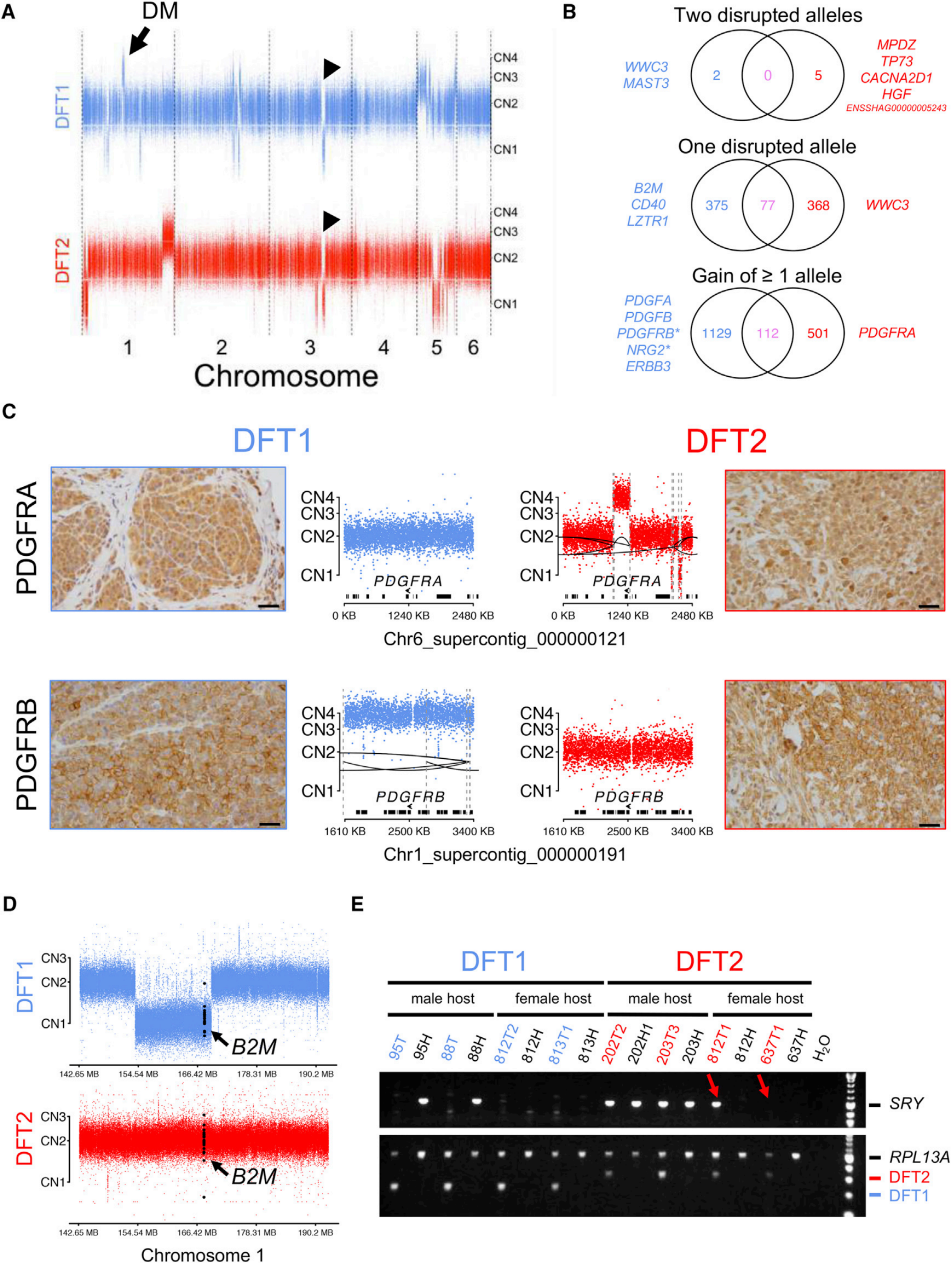
Copy Number Variation and Functional Annotation in DFT1 and DFT2 (A) DFT1 (tumor 88T) and DFT2 (tumor 202T2) autosomal copy number generated using read counts at 735,281 SNP loci. Each dot represents log_2_R, where R = (read depth in tumor)/(read depth in 203H), at a single SNP. CN, copy number. Arrow, chromosomal origin of DMs; arrowheads, hemizygous deletion identified in both DFT1 and DFT2. (B) Illustration of gene alterations. In each Venn diagram, number of genes altered in ≥1 DFT1 tumors are shown in blue on the left, and number of genes altered in ≥1 DFT2 tumors are shown in red on the right; numbers of genes that are similarly altered in ≥1 DFT1 and ≥1 DFT2 tumor are shown in pink in the center of each diagram. Only autosomal genes are considered and ‘disrupted’ alleles include only predicted loss-of-function alterations. Genes-of-interest are written in text beside diagrams. ^∗^ indicates these genes are amplified on extrachromosomal DMs. (C) Copy number and immunohistochemistry for *PDGFRA* and *PDGFRB*. Reads mapping within 500 base pair genomic bins were counted and normalized using cn.MOPS (Klambauer et al., 2012); each dot represents log_2_R for a single bin, where R = (read count tumor)/(read count 203H). CN, copy number. Structural variants are represented by dashed gray lines connected by black lines. Genes are represented as black bars, and locations and orientations of *PDGFRA* and *PDGFRB* are shown. Brown stain reports expression, counterstained with blue hematoxylin. Scale bar, 30 μm. (D) Copy number at *B2M* locus. Copy number was determined and displayed as in (C). Bins within *B2M* are colored in black. CN, copy number. (E) PCR amplification of the Y chromosome-linked *SRY* locus. DFT1 tumors (95T, 88T, 812T2, and 813T1) and DFT2 tumors (202T2, 203T3, 812T1, and 637T1) are labeled in blue and red, respectively, and DFT1 hosts (95H, 88H, 812H, and 813H) and DFT2 hosts (202H1, 203H, 812H, and 637H) are displayed in black. The upper panel shows *SRY* product and the lower panel shows positive control (*RPL13A*) and diagnostic amplification product for confirmation of DFT1 or DFT2 (Kwon et al., 2018). Red arrows highlight presence (812T1) or absence (637T1) of an *SRY* band in DFT2 tumors infecting female Tasmanian devils. See also Tables S3, S4, S5, S6, and Figure S2.

We next characterized copy number variants (CNVs) in the two cancers. A comparison of CNVs in DFT1 and DFT2 confirmed that all of the tumor isolates analyzed here are largely diploid (Figure 4A; Table S4). Most CNVs in DFT1 and DFT2 involved different genomic regions; however, an ∼18.4 megabase hemizygous deletion on chromosome 3 was found in both lineages (Figure 4A; Table S4). This CNV, which was not detected in 46 normal devil genomes suggesting that it is possibly somatic (Figure S2), reduces dosage of 74 genes in both DFT1 and DFT2 (Figure 4B and Table S4). One gene in DFT1 (*MAST3*) (Murchison et al., 2012) and four genes in DFT2, including *HGF* and *TP73*, have undergone homozygous deletion (Figure 4B; Table S4); the other two homozygously deleted genes in DFT2, *CACNA2D1* and *ENSSHAG0000005243*, are linked to *HGF* and *TP73*, respectively. Interestingly, *TP73* acts downstream of Hippo pathway effectors to activate apoptosis (Moroishi et al., 2015).

Copy number gains have increased the dosage of 1,129 genes in DFT1 and 501 genes in DFT2. Strikingly, we observed that genes encoding the two platelet-derived growth factor receptors (PDGFRs), *PDGFRA* and *PDGFRB*, were respectively gained in copy number in DFT2 (copy number 4, focal amplification) and some DFT1s (as part of extrachromosomal DMs) (Figure 4C; Tables S3 and S4). This correlated with strong expression of both PDGFRs in DFT1 and DFT2 (Figure 4C). Interestingly, both *PDGFA* and *PDGFB*, encoding ligands for PDGFRs, have undergone copy number gains in DFT1 (and *PDGFA* is additionally involved in a SV in DFT1 [Murchison et al., 2012; Tables S4 and S5]). Furthermore, *ERBB3* showed copy number gains in DFT1 and is expressed in DFT1 (Hayes et al., 2017, Taylor et al., 2017), and a subset of DFT1s carried gains of *NRG2*, encoding an ERBB ligand (Figure 4B; Tables S3 and S4).

### Immune Genes and Loss of Y Chromosome

DFT clones must escape the host immune system despite their status as allogeneic grafts. Interestingly, *B2M*, encoding a component of MHC class I, has undergone hemizygous deletion in DFT1 (Figure 4D). This copy number loss may have contributed to the downregulation of MHC observed in DFT1, resulting in this lineage's low immunogenicity (Siddle et al., 2013). We also observed that DFT1 carried a heterozygous truncating mutation in *CD40*, encoding an immune co-stimulatory molecule that may be expressed together with MHC class II by Schwann cells (Figures 2C and 4B) (Duan et al., 2007, Meyer zu Hörste et al., 2010).

DFT2 faces a further potential immunological challenge due to its possession of the Y chromosome. This lineage arose in a male devil and has, to date, usually been observed in males (of the 11 reported cases of DFT2, 9 involve a male host [Kwon et al., 2018]). This apparent bias toward male hosts raises the possibility that females may be less susceptible to DFT2 due to immunogenicity of antigens derived from the Y chromosome. We investigated the stability of the Y chromosome in DFT2 by PCR amplifying the Y-linked *SRY* locus in a panel of DFT tumors and their male and female hosts (Figure 4E; Table S6). As expected, Y chromosome DNA was not detected in DFT1, which is derived from a female founder devil, regardless of the gender of the host (Figure 4E). In DFT2, Y chromosome DNA was present in DFT2 tumors in male hosts, as well as in one DFT2 tumor in a female host, Devil 812 (Devil 812 also carried two DFT1 tumors [Kwon et al., 2018]). However, the Y chromosome locus could not be detected in the DFT2 tumor derived from the second female host, Devil 637 (Figure 4E).

### DFT1 and DFT2 Drug Screen

**Figure 5:**
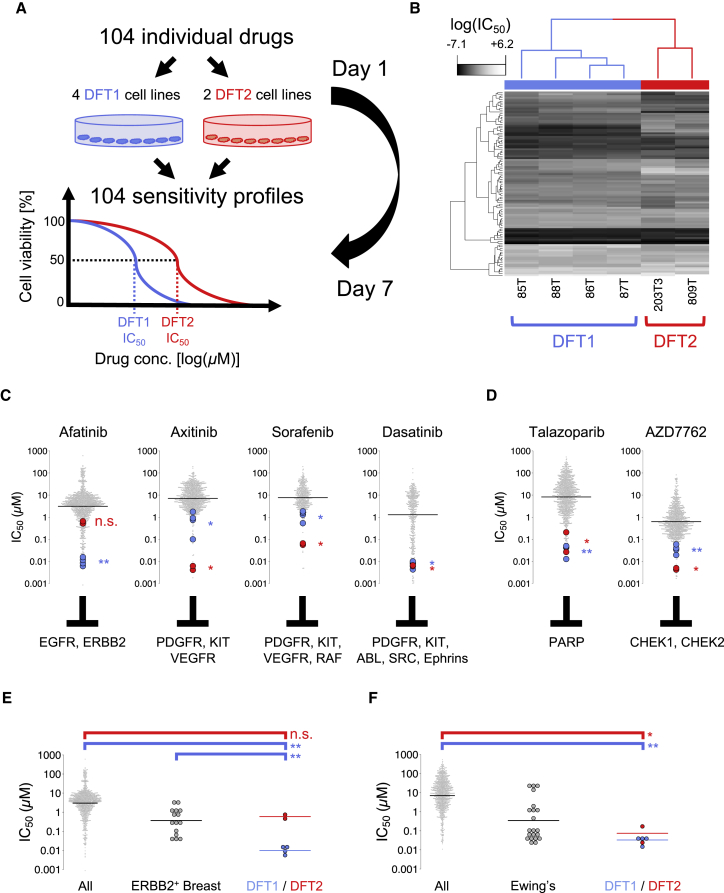
DFT1 and DFT2 Drug Screen (A) Four DFT1 (85T, 86T, 87T, and 88T) and two DFT2 (203T3 and 809T) cell lines were screened against a panel of 104 drugs under clinical and pre-clinical investigation in human oncology. Cell viability was measured after 144 hr. (B) Hierarchical clustering of log_*e*_ (IC_50_) values for 6 DFT cell lines (columns) screened with 104 compounds (rows). (C and D) IC_50_ for DFT1 (blue) and DFT2 (red) cell lines for four receptor tyrosine kinase (RTK) inhibitors (C) or two DNA repair inhibitors (D). Gray dots represent human cancer cell lines (GDSC set). Drug molecular targets are indicated. Horizontal bars represent geometric mean IC_50_. ^∗^p < 0.05, ^∗∗^p < 0.001; n.s., not significant, Wilcoxon rank-sum test for DFT1 and DFT2 compared with human cell lines. (E) Afatinib IC_50_ for 953 human cancer cell lines (All), 15 *ERBB2*-amplified human breast cancer cell lines (ERBB2^+^ breast), and DFT1 and DFT2 cell lines. Horizontal bars represent geometric mean IC_50_. ^∗∗^p < 0.001; n.s., not significant, Wilcoxon rank-sum test. (F) Talazoparib IC_50_ for 922 human cancer cell lines (All), 21 Ewing's sarcoma cell lines (Ewing's), and DFT1 and DFT2 cell lines. Horizontal bars represent geometric mean IC_50_. ^∗^p < 0.05, ^∗∗^p < 0.001; Wilcoxon rank-sum test. See also Table S7.

To gain further insight into the signaling pathways which promote DFT1 and DFT2 growth and survival, and to uncover potential therapeutic vulnerabilities, we performed a high-throughput *in vitro* drug sensitivity screen. Four DFT1 cell lines and two DFT2 cell lines (Table S7) were treated with a 7-point titration (1,000-fold concentration range) of 104 pre-clinical and clinical compounds with activity against a wide range of molecular targets (Figure 5A; Table S7) prior to cell viability quantification. Hierarchical clustering based on half maximal inhibitory concentration (IC_50_) values indicated that DFT1 and DFT2 are distinguishable from each other based on their drug sensitivity (Figure 5B); however, the two cancers share a striking overall similarity in drug response profile compared with several hundred human cancer cell lines (Figures 5C–5F; Table S7 [Yang et al., 2013]).

Both DFT1 and DFT2 are sensitive to a suite of inhibitors of receptor tyrosine kinases (RTKs) (Figure 5C). In particular, DFT1 cell lines are remarkably responsive to Afatinib, an inhibitor of ERBB2 and EGFR (DFT1 cell lines top 0.4%–1.1% most sensitive of 959 cell lines, geometric mean DFT1 IC_50_: 9.8 nM) (Figure 5C). This sensitivity is likely mediated by ERBB2 inhibition, as DFT1 is resistant to Gefitinib and Erlotinib, agents that specifically target EGFR (Table S7). Remarkably, DFT1 cell lines show significantly greater sensitivity to Afatinib than a panel of *ERBB2*-amplified human breast cancer cell lines (Figure 5E; geometric mean DFT1 IC_50_: 9.8 nM, geometric mean ERBB2^+^ Breast cancer cell lines IC_50_: 314.9 nM; p = 0.000516, Wilcoxon rank-sum test). DFT2, on the other hand, is highly sensitive to Axitinib, a compound with activity against PDGFR, KIT, and vascular endothelial growth factor receptor (VEGFR) (DFT2 cell lines top 0.2%–0.4% most sensitive of 854 cell lines, geometric mean DFT2 IC_50_: 5.0 nM) (Figure 5C). In addition, both DFT1 and DFT2 show sensitivity to Dasatinib, a tyrosine kinase inhibitor whose targets include PDGFR, ABL, SRC, ephrins, and KIT (geometric mean DFT1 IC_50_: 7.5 nM; geometric mean DFT2 IC_50_: 6.4 nM) (Figure 5C). Both DFT1 and DFT2 are markedly sensitive to CHEK1/CHEK2 inhibitor AZD7762, and poly-ADP ribose polymerase (PARP) inhibitors Talazoparib and Olaparib, suggesting that DFT cancers are intolerant of DNA damage (Figure 5D; Table S7), perhaps explaining the remarkable genomic stability observed in DFT1 (Deakin et al., 2012, Murchison et al., 2012). The response of DFT cell lines to Talazoparib was particularly notable and is comparable with that of highly sensitive human Ewing's sarcoma cell lines (geometric mean DFT1 IC_50_: 33.1 nM, top 0.2%–2.0% of 922 cell lines, geometric mean DFT2 IC_50_: 74.2 nM, top 0.7%–5.2% of 922 cell lines, geometric mean Ewing's IC_50_: 330.2 nM, top 0.3%–72.1% of 922 cell lines) (Brenner et al., 2012, Garnett et al., 2012) (Figure 5F). This sensitivity likely does not reflect defects in homologous recombination, as we do not detect evidence for COSMIC mutational signature 3 (Figure 2A; Table S2) (Alexandrov et al., 2015b, Alexandrov et al., 2013, COSMIC, 2017, Nik-Zainal et al., 2012, Nik-Zainal et al., 2016). Altogether, this screen highlights key vulnerabilities inherent to DFT cells and strongly implicates RTK signaling in driving oncogenesis of both DFT1 and DFT2.

## Discussion

DFT2 has changed our perception of the nature of transmissible cancers. Previously, transmissible cancers were believed to arise very rarely in nature, with the existing examples representing exceptional cases that had overcome strong natural barriers. Indeed, the observation that all sampled transmissible venereal tumors in dogs belong to a single clone which originated several thousand years ago (Murgia et al., 2006, Rebbeck et al., 2009, Strakova et al., 2016) suggests that such canine cancers appear and disperse infrequently. However, the emergence of DFT2, together with the discovery of several transmissible cancers in marine bivalves (Metzger et al., 2015, Metzger et al., 2016), suggests that some species may have a particular vulnerability for the development of this type of disease and that, at least in these species, transmissible cancers may be spawned relatively frequently.

The reason for Tasmanian devils' apparent susceptibility to transmissible cancers is not clear. The striking similarities in tissues-of-origin, genome architectures, mutational processes, driver gene candidates, and drug vulnerabilities, strongly suggest that DFT1 and DFT2 belong to the same cancer type and arose via similar oncogenic mechanisms. DFTs are likely of neuroectodermal origin, and may show differentiation toward the neural crest-derived Schwann cell lineage (Murchison et al., 2010). The closest human cancer histotype to DFT is not clear (Loh et al., 2006b), and comparative studies with human and veterinary cancers are further hampered by lack of knowledge of the body site from which DFT cancers first arise. It is notable that, although Tasmanian devils are reported to have high frequencies of host-derived neoplasia (Griner, 1979), no lesions have been described that are consistent with pre-transmissible DFT; given that hundreds of wild and captive devils are routinely monitored each year, this suggests that either such lesions are difficult to detect or recognize or that DFT cancers arise rarely but carry a high risk of becoming transmissible. Importantly, we cannot completely negate the possibility that DFT1 and/or DFT2 arose via a horizontal DNA transfer event involving an ancestral DFT cell and a normal cell (Pye et al., 2016b); however, the lack of germline and somatic genetic similarity between DFT1 and DFT2 suggests that this scenario is unlikely.

We investigated genetic and phenotypic features of DFT1 and DFT2, and compared the two lineages with each other and with catalogs of known human cancer genes and drug sensitivity profiles. These data suggest an important role for RTK signaling, most likely involving ERBB2 (DFT1 only) and PDGFRs (DFT1 and DFT2), in sustaining growth and survival of DFT cancers. In this context, it is likely that copy number gains involving PDGFR genes may have provided selective advantage in these cancers. Furthermore, we noted that *PDGFRB* has been amplified on DMs in some DFT1s, and may be the positively selected driver required to maintain this extrachromosomal DNA. We did not identify any mutations in *ERBB2* in DFT1. However, we observed copy number gains involving *ERBB3*, encoding an ERBB2 heterodimerization partner, and *NRG2,* encoding an ERBB3 ligand (Hynes and Lane, 2005, Taylor et al., 2017), suggesting a possible mechanism for ERBB2 activation. Both DFTs show remarkably few perturbations in known cancer genes, and only two genes in DFT1 and five genes in DFT2 are predicted to have undergone biallelic loss-of-function. Thus, the observation that DFT1 and DFT2 both harbor predicted two-hit loss-of-function mutations in genes encoding proposed regulators and effectors of Hippo signaling (*WWC3, MPDZ, TP73*), together with evidence for activity of Hippo effectors YAP1 and WWTR1/TAZ in DFT1 and DFT2 cells, raises the possibility that this pathway is involved in DFT cancers in Tasmanian devils. The Hippo pathway plays conserved roles in differentiation, proliferation, and regeneration in several tissues (Moroishi et al., 2015, Yu et al., 2015, Zanconato et al., 2016), and in the Schwann cell context drives transcriptional upregulation of PDGF and ERBB signaling components (Deng et al., 2017, Wu et al., 2018).

DFT clones must escape the host immune system despite their status as allogeneic grafts. Although low Tasmanian devil population genetic diversity may reduce capacity for foreign tissue detection (Miller et al., 2011, Siddle et al., 2007), this species' rejection of skin allografts (Kreiss et al., 2011) suggests that DFT1 and DFT2 clones have specific adaptations favoring immune escape. Our analysis did not identify any genomic aberrations common to both cancers that might underlie such adaptations, raising the possibilities that they may be epigenetically controlled (Siddle et al., 2013), or that DFT cancers arise from cell types that already harbor low immunogenicity. Nevertheless, it is possible that hemizygous deletion of *B2M* may have contributed to downregulation of MHC class I in DFT1, although the remaining intact copy can be robustly expressed in response to the inflammatory cytokine, interferon gamma (Siddle et al., 2013). In DFT2, both copies of *B2M* remain intact, and *B2M* expression has been detected in at least a subset of tumor cells (H. Siddle, unpublished data). This suggests that DFT1 and DFT2 may have adopted different strategies for immune evasion, although the significance of these findings is not yet confirmed. Loss of Y chromosome DNA in DFT2 may have rendered this cancer less immunogenic in female hosts, although we cannot exclude the possibility that this loss is selectively neutral. If Y chromosome loss is indeed a selective advantage to the lineage, we may expect in future to observe Y-null DFT2 strains, perhaps derived from several independent Y chromosome loss events, becoming dominant in the population. Despite limited understanding of the mechanisms of DFT immune evasion, recent observations of natural immune responses against DFT1 (Pye et al., 2016a), as well as allele frequency shifts indicative of selection in DFT1-affected populations (Epstein et al., 2016), suggest that some devils may be capable of mounting immune responses against DFT cancers.

Altogether, our findings present the possibility that transmissible cancers may be a part of Tasmanian devils' natural ecology. Indeed, we did not find evidence for the involvement of exogenous exposures or pathogens in DFT carcinogenesis, nor did we identify any known cancer predisposition alleles in the inherited genomes of the DFT1 or DFT2 founder devils. Thus, it seems plausible that additional DFTs occurred in the past but escaped detection, perhaps because they remained in localized populations or because they existed prior to the nineteenth-century arrival of European documenters.

It is worth speculating about biological features specific to devils that may spur DFT cancer development. Devils bite each other frequently around the facial area, often causing significant tissue injury (Hamede et al., 2013). Given the important roles for Hippo and RTK signaling in wound-healing responses (Zanconato et al., 2016), particularly in Schwann cells (Mindos et al., 2017, Fex Svennigsen and Dahlin, 2013), it is tempting to speculate that DFT cancers may arise from aberrant maintenance of proliferative cells involved in tissue repair after injury. Under this model, the facial biting behavior of Tasmanian devils may predispose these animals to emergence of cancers via tissue injury, simultaneously providing a route of cell transmission. Furthermore, it is possible that anthropogenic factors may have indirectly increased the risk of DFT emergence or spread in recent years. For instance, it is possible that some modern land use practices, such as pastoralism, may have provided favorable conditions for devils, leading to elevations in local devil densities (Guiler, 1970, Guiler, 1982, Jones et al., 2004); this might have led to increased intra-specific competition, perhaps increasing interactions and fights, which may in turn have raised probabilities of DFTs arising or spreading. Road construction may have caused increased connectivity between devil populations, providing more opportunities for DFTs to spread. Finally, persecution of devils by European colonists (Hawkins et al., 2006) may have contributed to this species' low genetic diversity (Jones et al., 2004), a possible risk factor for DFT immune escape and disease spread (Siddle et al., 2007). In future, it will be important to continue to monitor Tasmanian devils for evidence of additional DFT clones and to track the evolution and spread of DFT1 and DFT2.

At present, there are few options for DFT treatment, and most animals succumb to disease. Given the failure of conventional chemotherapy agents against DFT1 (Phalen et al., 2013), the potential for orally delivered, targeted therapies offer considerable promise. We have shown that DFT1 and DFT2 are exquisitely sensitive to a suite of RTK inhibitors, including those targeting PDGFRs (DFT1 and DFT2) and ERBB2 (DFT1 only), as well as to inhibitors of DNA repair. The recent success of experimental immunotherapy regimens against DFT1 (Tovar et al., 2017) opens the possibility that therapies which combine RTK or PARP inhibition with immune activation may present new opportunities for combatting DFT clones and saving the Tasmanian devil.

DFT1 and DFT2 arose from two unremarkable individuals, which gave rise to cancers with strikingly similar, but subtly different, histologic, genomic, and drug sensitivity phenotypes. We have shown that, at least in Tasmanian devils, relatively simple genomic changes coupled with incessant growth factor signaling spur the transition from somatic cell to parasitic clonal lineage. Transmissible cancers in Tasmanian devils exploit a perverse niche created by their host species and illustrate one context in which runaway selfish evolution can thrive.

## STAR★Methods

### Key Resources Table

**Table:** 

REAGENT or RESOURCE	SOURCE	IDENTIFIER
**Antibodies**		

Monoclonal mouse anti-human Cytokeratin	Dako	Cat# M3515; RRID: AB_2132885
Monoclonal mouse anti-human Muscle Specific Actin	Leica Microsystems	Cat# NCL-MSA; RRID: AB_563409
Monoclonal mouse anti-human Neuron Specific Enolase	Dako	Cat# M0873; RRID: AB_2099322
Monoclonal mouse anti-human Smooth Muscle Antigen	Dako	Cat# M0851; RRID: AB_2223500
Monoclonal mouse anti-human Vimentin	Dako	Cat# M0725; RRID: AB_10013485
Monoclonal mouse anti-human YAP1	Sigma-Aldrich	Cat# WH0010413M1; RRID: AB_1844253
Monoclonal rabbit anti-human PDGFRB	Abcam	Cat# ab32570; RRID: AB_777165
Polyclonal rabbit anti-human PDGFRA	Abcam	Cat# ab124392; RRID: AB_10978090
Polyclonal rabbit anti-human Periaxin	Sigma-Aldrich	Cat# HPA001868; RRID: AB_2172440
Polyclonal rabbit anti-human S100	Dako	Cat# Z0311; RRID: AB_10013383
Polyclonal rabbit anti-human WWTR1/TAZ	Sigma-Aldrich	Cat# T4077; RRID: AB_1841213

**Biological Samples**		

Devil facial tumor disease 1 (DFT1) biopsy: 36T2	This paper	Table S2
Devil facial tumor disease 1 (DFT1) biopsy: 96T	This paper	Table S2
Devil facial tumor disease 1 (DFT1) biopsy: 221T	This paper	Table S2
Devil facial tumor disease 1 (DFT1) biopsy: 331T	This paper	Table S2
Devil facial tumor disease 1 (DFT1) biopsy: 333Ta	This paper	Table S2
Devil facial tumor disease 1 (DFT1) biopsy: 812T2	This paper	Table S6
Devil facial tumor disease 1 (DFT1) biopsy: 813T1	This paper	Table S6
Devil facial tumor disease 2 (DFT2) biopsy: 637T1	This paper	Table S6
Tasmanian devil buffy coat: 95H	This paper	Table S6
Tasmanian devil buffy coat: 124H	This paper	Table S2
Tasmanian devil ear biopsy: 122H1	This paper	Table S2
Tasmanian devil ear biopsy: 133H	This paper	Table S2
Tasmanian devil ear biopsy: 238H	This paper	Table S2
Tasmanian devil ear biopsy: 244H	This paper	Table S2
Tasmanian devil ear biopsy: 264H	This paper	Table S2
Tasmanian devil ear biopsy: 265H	This paper	Table S2
Tasmanian devil ear biopsy: 266H	This paper	Table S2
Tasmanian devil ear biopsy: 267H	This paper	Table S2
Tasmanian devil ear biopsy: 268H	This paper	Table S2
Tasmanian devil ear biopsy: 269H	This paper	Table S2
Tasmanian devil ear biopsy: 270H	This paper	Table S2
Tasmanian devil ear biopsy: 270H	This paper	Table S2
Tasmanian devil ear biopsy: 317H	This paper	Table S2
Tasmanian devil ear biopsy: 637H	This paper	Table S6
Tasmanian devil ear biopsy: 811H	This paper	Table S2
Tasmanian devil ear biopsy: 812H	This paper	Table S6
Tasmanian devil ear biopsy: 813H	This paper	Table S6
Tasmanian devil kidney biopsy: 203H	Pye et al., 2016b	SN-H; Tables S1 and S2
Tasmanian devil liver biopsy: 31H	Murchison et al., 2012	Male normal devil; Table S2
Tasmanian devil liver biopsy: 63H1	This paper	Table S2
Tasmanian devil liver biopsy: 110H	This paper	Table S2
Tasmanian devil liver biopsy: 112H	This paper	Table S2
Tasmanian devil liver biopsy: 115H1	This paper	Table S2
Tasmanian devil liver biopsy: 117H	This paper	Table S2
Tasmanian devil liver biopsy: 119H	This paper	Table S2
Tasmanian devil liver biopsy: 134H1	This paper	Table S2
Tasmanian devil liver biopsy: 347H	This paper	Table S2
Tasmanian devil liver biopsy: 379H	This paper	Table S2
Tasmanian devil liver biopsy: 420H	This paper	Table S2
Tasmanian devil liver biopsy: 442H	This paper	Table S2
Tasmanian devil liver biopsy: 443H	This paper	Table S2
Tasmanian devil liver biopsy: 444H	This paper	Table S2
Tasmanian devil spleen biopsy: 11H1	This paper	Table S2
Tasmanian devil spleen biopsy: 202H1	Pye et al., 2016b	RV-H; Tables S1 and S2
Tasmanian devil spleen biopsy: 398H	This paper	Table S2

**Chemicals, Peptides, and Recombinant Proteins**		

Chemotherapeutic Compounds List	This paper	Table S7
Syto60	Thermo Fisher Scientific	Cat# S11342

**Critical Commercial Assays**		

Agilent SureSelect XT, HSQ	Agilent Technologies	Cat# G9611A
DNeasy Blood and Tissue kit	Qiagen	Cat# 69504
EZ-PCR Mycoplasma Test	Biological Industries	Cat# 20-700-20
Fluorescence-based live-cell assay	Thermo Fisher Scientific	Cat# L324
Genomic-Tip kit	Qiagen	Cat# 10223
illustra GenomiPhi V2 DNA Amplification kit	GE Healthcare	Cat# 25660030
MycoAlert Mycoplastma Detection kit	Lonza	Cat# LT07-118
NEBNext Sanger Sequencing Kit for MiSeq libraries	NEB	Cat# E7645S
QIAquick PCR Purification kit	Qiagen	Cat# 28106
Telomere PNA kit	Dako	Cat# K5327

**Deposited Data**		

Aligned sequencing reads of tumors and normals	This paper	ENA: PRJEB21902
Aligned sequencing reads of 12 normals from West Pencil Pine	Wright et al., 2017	ENA: PRJEB8782
COSMIC Cancer Gene Census	Forbes et al., 2015	http://cancer.sanger.ac.uk/census/
COSMIC consensus mutational signatures	Alexandrov et al., 2013	http://cancer.sanger.ac.uk/cosmic/signatures
Drug Sensitivity in Cancer (GDSC) IC50 data	Yang et al., 2013	http://www.cancerrxgene.org/downloads
RAD Sequencing data of 527 Tasmanian devils	Brüniche-Olsen et al., 2016	http://datadryad.org/resource/doi:10.5061/dryad.86bq5
Raw data	This paper	https://doi.org/10.17632/znfphvhmbv.1
Tasmanian devil reference genome 7.0	Murchison et al., 2012	https://www.ensembl.org/Sarcophilus_harrisii/
Tasmanian devil tumor and host contigs from de novo assemblies	This paper	ENA: PRJEB21902, https://doi.org/10.17632/znfphvhmbv.1

**Experimental Models: Cell Lines**		

Devil facial tumor disease 1 (DFT1): 85T	This paper	Tables S2 and S7
Devil facial tumor disease 1 (DFT1): 86T	Siddle et al., 2013	DFTD 1426; Tables S1, S2, S6, and S7
Devil facial tumor disease 1 (DFT1): 87T	Siddle et al., 2013	DFTD C5065; Tables S2 and S7
Devil facial tumor disease 1 (DFT1): 88T	Siddle et al., 2013	DFTD 4906; Tables S1, S2, S6, and S7
Devil facial tumor disease 1 (DFT1): 95T	This paper	Tables S2 and S6
Devil facial tumor disease 2 (DFT2): 202T2	Pye et al., 2016b	RV-T; Tables S1, S2, and S6
Devil facial tumor disease 2 (DFT2): 203T3	Pye et al., 2016b	SN-T; Tables S1, S2, S6, and S7
Devil facial tumor disease 2 (DFT2): 338T	Pye et al., 2016b	JV-T; Table S2
Devil facial tumor disease 2 (DFT2): 339T	Pye et al., 2016b	NR-T; Table S2
Devil facial tumor disease 2 (DFT2): 809T1	This paper	Table S7
Tasmanian devil fibroblasts: 91H	Murchison et al., 2012	Female normal devil, reference animal; Table S2

**Oligonucleotides**		

DFT diagnostic oligos	Kwon et al., 2018	N/A
*MPDZ* forward: 5'-GGT CTT GGA TGA ACA AAA GAA GA-3'	This paper	N/A
*MPDZ* reverse: 5'-ACT GTA CGG CTG GCA CTG AT-3'	This paper	N/A
*PDZD11-RFX2* forward: 5'- ACC GCC AAG TTT CAA ATC AG-3'	This paper	N/A
*PDZD11-RFX2* reverse: 5'- TCC TCC AGG ATA CCT CTC CA-3'	This paper	N/A
Single Nucleotide Variant (SNV) Validation oligos	This paper	Table S2
*SRY* forward: 5'-GCG ACC GTT CAT TGA CGA AG-3'	This paper	N/A
*SRY* reverse: 5'-ACA GAT TTG GGG ACA CGA GG-3'	This paper	N/A
Structural Variant (SV) Validation oligos	This paper	Table S5
Tasmanian devil chromosome-specific FISH probes	Murchison et al., 2012	N/A
*WWC3* forward: 5'-CAA AAA CTA AAG CAA AAA CCA AGA-3'	This paper	N/A
*WWC3* reverse: 5'-CCA GAA GGC CTA TTG AAT TCC T-3'	This paper	N/A

**Software and Algorithms**		

alleleCount	Cancer Genome Project, Wellcome Trust Sanger Institute	https://github.com/cancerit/alleleCount
Breakpoints via Assembly (BRASS)	Cancer Genome Project, Wellcome Trust Sanger Institute	https://github.com/cancerit/BRASS
BWA-backtrack	Li and Durbin, 2009	http://bio-bwa.sourceforge.net/
BWA-MEM	Li, 2013	http://bio-bwa.sourceforge.net/
Fermi	Li, 2012	https://github.com/lh3/fermi
Integrative Genomics Viewer (IGV)	Thorvaldsdóttir et al., 2011	http://software.broadinstitute.org/software/igv/
Phusion2	Mullikin and Ning, 2003	http://www.sanger.ac.uk/science/tools/phusion2
PICARD	DePristo et al., 2011	http://broadinstitute.github.io/picard/
Platypus	Rimmer et al., 2014	http://www.well.ox.ac.uk/platypus
R Language and Environment for Statistical Computing	R Core Team, 2015	https://www.R-project.org/
R Bioconductor Suite	Huber et al., 2015	https://www.bioconductor.org/
R circlize package	Gu et al., 2014	https://github.com/jokergoo/circlize
R cn.MOPS package	Klambauer et al., 2012	http://www.bioinf.jku.at/software/cnmops/
R Stan interface	Carpenter et al., 2017	http://mc-stan.org/users/interfaces/rstan
Samtools	Li et al., 2009	http://samtools.sourceforge.net/
scanPAV	Giordano et al., 2018	https://github.com/wtsi-hpag/scanPAV
SOAPdenovo	Li et al., 2010	http://soap.genomics.org.cn/soapdenovo.html
Somatypus (cancer data adaptation of Platypus)	This paper	https://github.com/baezortega/somatypus
SSPACE	Boetzer et al., 2011	https://www.baseclear.com/genomics/bioinformatics/basetools/SSPACE
SvABA	Wala et al., 2017	https://github.com/walaj/svaba
TIGRA Assembler	Chen et al., 2014	http://bioinformatics.mdanderson.org/main/TIGRA
Variant Effect Predictor (VEP)	McLaren et al., 2010	https://www.ensembl.org/info/docs/tools/vep/index.html

**Other**		

Custom R scripts for data analysis and reproduction	This paper	https://github.com/MaximilianStammnitz

### Contact for Reagent and Resource Sharing

Further information and requests for resources and reagents should be directed to and will be fulfilled by the lead contact, Elizabeth Murchison (epm27@cam.ac.uk). Tasmanian devil material can only be shared with the permission of the Tasmanian Government.

### Experimental Model and Subject Details

#### Tissue Sampling and Ethics

Tissues were sampled from wild Tasmanian devils that were subsequently released, or from animals euthanized for welfare reasons. All animal procedures were performed under a Standard Operating Procedure approved by the General Manager, Natural and Cultural Heritage Division, Tasmanian Government Department of Primary Industries, Parks, Water and the Environment (DPIPWE), in agreement with the DPIPWE Animal Ethics Committee, or under University of Tasmania Animal Ethics Committee Permit A0014976. The project was approved by the University of Cambridge Department of Veterinary Medicine Ethics and Welfare Committee, reference CR191.

#### Cell Lines and Cell Culture

DFT1 cell lines 86T and 88T have been previously described with the names 1426 and 4906 respectively (Siddle et al., 2013). DFT2 cell lines 202T2 and 203T3 cell lines were established as follows. Micro-biopsies of approximately 2 mm in diameter were collected into RPMI 1640 (Thermo Fisher Scientific, Waltham, MA, USA) with 2% vol./vol. antibiotic-antimycotic (Thermo Fisher Scientific). Biopsies were flushed through a tea-strainer sized metal mesh with amniomax (Thermo Fisher Scientific). Subsequently, cells were plated in 6 well flat-bottomed plates (Corning Inc., Corning, NY, USA) with 3 ml amniomax and 2% vol./vol. antibiotic-antimycotic, and placed at 35°C with 5% atm. CO_2_. After 24 hr, medium was replaced and plates were incubated with the same conditions for an additional 48 hr. Cells were then transferred into T25 flasks with the same medium, and after reaching confluence approximately 48 hr later, flasks were split and media changed to RPMI 1640; 1% vol./vol. GlutaMAX (Thermo Fisher Scientific); 10% vol./vol. FCS (Bovogen Biologicals, Melbourne, VIC, Australia), 20% vol./vol. amniomax and 1% vol./vol. antibiotic-antimycotic. We used MycoAlert (Lonza, Basel, Switzerland) and EZ-Mycoplasma Test (Biological Industries, Kibbutz Beit-Haemek, Israel) kits to screen cell lines for Mycoplasma according to manufacturers’ instructions. Details about dates of sampling and Mycoplasma status for cell lines sequenced in this study are indicated below.

**Table:** 

Name	Year of Establishment	Year of DNA Extraction	Estimated Tumor Purity∗	Mycoplasma
86T	2005	2009	100%	negative
88T	2007	2009	100%	negative
202T2	2014	2015	100%	positive
203T3	2014	2015	90-95%	negative

∗ See *SNV-based Tumor Purity Estimation* for methods.

Tables S1, S2, S6, and S7 and the Key Resources Table list information on all Tasmanian devil and DFT cell lines, as well as other samples used in this study.

### Method Details

#### Cytogenetics

Chromosome-specific probes were derived from flow sorted chromosomes and hybridized with metaphases as described (Murchison et al., 2012). For fluorescence *in situ* hybridization with telomeric probes, we used the Telomere PNA (Peptide Nucleic Acids)/Cy3 kit (Dako, Glostrup, Denmark). There are two nomenclature systems in use for Tasmanian devil chromosomes (Deakin et al., 2012, Pearse and Swift, 2006). These two systems differ in their designations of the two largest devil chromosomes, chromosomes 1 and 2. The chromosome named chromosome 1 in the first system is named chromosome 2 in the second system, and vice versa. In this study, we used the nomenclature adopted by Pearse and Swift (Pearse and Swift, 2006); this system is also used in the Tasmanian devil reference genome (Murchison et al., 2012).

#### Histology

Tasmanian devil tissues were fixed in 10% Neutral Buffered Formaldehyde (Australian Biostain, Traralgon, VIC, Australia) for 24 hr and selected tissues were cassetted (Techno Plas, St. Marys, SA, Australia) and processed overnight using a standard 15-hr overnight procedure in the TP 1050 tissue processor (Leica Microsystems, Wetzlar, Germany). Tissues were orientated on the EG1160 (Leica Microsystems), embedded in paraffin wax (Leica Microsystems) and sectioned at 3 microns using a Leica RM2245 microtome and adhered to microscope slides (Menzel Gläser, Thermo Fisher Scientific) for 20 min at 60°C. Sections were deparaffinized, rehydrated and stained using Jung autostainer XL (Leica Microsystems) for Hematoxylin (Australian Biostain) and Eosin, dehydrated, cleared, cover slipped (Leica Microsystems) and mounted in CV Mount (Leica Microsystems) (Hayes et al., 2017).

#### Immunohistochemistry

Tasmanian devil tissues and tumors were sectioned at 3 microns, floated onto Superfrost plus slides (Menzel Gläser) and subjected to standard deparaffinization and rehydration techniques using an automated stainer (Leica Microsystems). Antigen retrieval in tissue sections was conducted in citrate buffer at pH 6.0 (Reveal Decloaker, Biocare Medical, Pacheco, CA, USA) at 120°C for 8 min using a Pascal pressure chamber (Dako) then cooled to 20°C. Endogenous peroxidase activity was quenched using 3% hydrogen peroxide (Ajax Finechem, Thermo Fisher Scientific) in methanol (Ajax Finechem) for 30 min. Detection of primary antibodies was achieved using Mach1 Universal HRP-Polymer detection kit (Biocare Medical). Protein block (Background Sniper, Biocare Medical) was applied for 20 min prior to application of primary antibodies. Polyclonal rabbit anti-human PDGFRA 1:800 (Cat#ab124392, Abcam, Cambridge, UK), Monoclonal rabbit anti-human PDGFRB 1:50 (Cat#ab32570, Abcam), Polyclonal Rabbit anti-human S100 1:1500 (Cat#Z0311, Dako), Monoclonal Mouse anti-human Neuron Specific Enolase 1:200 (Cat#M0873, Dako), Monoclonal Mouse anti-human Cytokeratin 1:100 (Cat#M3515, Dako), Monoclonal Mouse anti-human Vimentin 1:800 (Cat#M0725, Dako), Monoclonal Mouse anti-human Smooth Muscle Antigen 1:200 (Cat#M0851, Dako), Monoclonal Mouse anti-human Muscle Specific Actin 1:50 (Cat#NCL-MSA, Leica Microsystems), Polyclonal Rabbit anti-human Periaxin 1:400 (Cat#HPA001868, Sigma-Aldrich, St. Louis, MO, USA), Monoclonal mouse anti-human YAP1 1:100 (Cat#WH0010413M1, Sigma-Aldrich) and Polyclonal rabbit anti-human WWTR1/TAZ 1:100 (Cat#T4077, Sigma-Aldrich) were diluted as indicated with antibody diluent (Dako) and applied to both devil tumor and normal devil control tissues at room temperature for 30 min. Negative control was omission of primary antibody with buffer substitution. Universal HRP-polymer was applied for 30 min (MRH538L10, Biocare Medical) followed by 1 drop of Betazoid DAB Chromogen 3,3 Diaminobenzidine (BDB900G, Biocare Medical) in 1 ml of substrate buffer (DB900, Biocare Medical) applied for 4 min. Tris-buffered saline (Biocare Medical) was used to rinse between all steps. Slides were rinsed, stained with Carazzi’s Hematoxylin for 5 min, washed for 3 min in tap water, dehydrated, cleared, cover slipped (CV5030, Leica Microsystems) and mounted in CV mount (Leica Microsystems) (Hayes et al., 2017). Sections were viewed under light microscopy using Olympus BX41 (Olympus Corporation, Shinjuko, Tokyo, Japan) and selected areas were photographed using a digital camera (DP20, Olympus Corporation).

#### Sample Processing and Sequencing

##### DNA Extraction

DNA from all samples except for 86T and 88T was extracted using the DNeasy Blood and Tissue extraction kit (Qiagen, Hilden, Germany). DNA from 86T and 88T was extracted using the Genomic-Tip kit (Qiagen).

##### Library Preparation

500 ng of genomic DNA was fragmented (average size distribution 425 base pair (BP), LE220, Covaris, Woburn, MA, USA), purified, libraries prepared (Agilent SureSelect XT, HSQ, Agilent Technologies, Santa Clara, CA, USA), and index tags applied (Sanger 168 tag set). Index tagged samples were amplified (6 cycles of PCR, KAPA HiFi kit, KAPA Biosystems, Wilmington, MA, USA), quantified (1k assay, LabChip GX, PerkinElmer, Waltham, MA, USA), then pooled together in an equimolar fashion.

##### High-Coverage DNA Sequencing

Pooled samples were quantified (1K assay, Bioanalyzer, Agilent Technologies), normalized (∼6 nM), and submitted to cluster formation for HiSeq V4 sequencing (125 BP paired-end (PE) reads, Illumina, San Diego, CA, USA). We sequenced the equivalent of two lanes per tumor, and one lane per host; however, sequencing was multiplexed across several lanes. The table below indicates average insert size, read length and average read depth for samples sequenced at high coverage in this study (see also Table S1).

**Table:** 

ID	Average Sequencing Depth	Average Insert Size	Read Length
202H1	49 X	417 BP	125 PE
202T2	67 X	418 BP	125 PE
203H	45 X	428 BP	125 PE
203T3	70 X	429 BP	125 PE
86T	86 X	430 BP	125 PE
88T	67 X	428 BP	125 PE

Sequence reads were aligned to the Tasmanian devil reference genome Devil7.1, an in-house assembly which is identical to the publicly available Devil7.0 (http://www.ensembl.org/Sarcophilus_harrisii/Info/Index), except Devil7.1 excludes the mitochondrial contig. Throughout the study, we used custom scaffold identifiers. Correspondence between our scaffold identifiers and those used in Devil7.0 can be found at Mendeley Data (https://doi.org/10.17632/znfphvhmbv.1). Alignment was performed using BWA-backtrack (Li and Durbin, 2009) and duplicate flagging and removal was conducted using PICARD (DePristo et al., 2011).

##### Low-Coverage DNA Sequencing

Thirty normal genomes were additionally sequenced at low coverage (∼1 X) (Table S2). Library preparation and sequencing were performed as described for high-coverage genomes. Reads were aligned to Devil7.1+MT with BWA-MEM.

#### Published Normal Devil Genomes

We included data from two previously sequenced normal Tasmanian devil genomes, 31H and 91H, in this study ((Murchison et al., 2012); 31H and 91H are the “male” and “female” normal genomes respectively). However, only a subset of 31H data (lanes 999#1, 999#2, 999#3, 999#4, 999#6, 1000#1, 1000#2, 1000#4, 1000#6, 1000#7, 1000#8, 1002#1, 1002#7, 1003#1, 1003#2, 1003#3, 1003#7) were included, as some lanes fell below sequencing quality thresholds (average sequencing coverage for this sample was ∼17 X). Two previously sequenced DFT1 tumors from this study, 53T and 87T (Murchison et al., 2012), were not included in the current study, as they fell below sequencing quality thresholds. Twelve previously sequenced devil normal genomes were also used in this study (Wright et al., 2017) (Table S2). These were aligned to Devil7.1+MT with BWA-MEM.

#### Whole Genome Amplification

Whole genome amplification was performed to create DNA stocks for PCR screening. Depending on the concentration, 1-2 μl of DNA (concentration range ∼20 to ∼50 ng/μl) from each sample was used as input for whole genome amplification using the illustra GenomiPhi V2 DNA Amplification kit (GE Healthcare, Chicago, IL, USA).

#### SNV Validation

We performed experimental validation on a set of 96 single-nucleotide variants (SNVs) obtained through our computational filtering pipelines. The SNVs selected for validation were derived from computation sets found in both DFT1s (86T and 88T), both DFT2s (202T2 and 203T3) or in all four tumors (86T, 88T, 202T2, 203T3). Primers were designed around each SNV (Table S2) and used to amplify a ∼500 BP region around the SNV site with conditions as follows. Template DNA was an equal volume pool of whole genome amplified DNA from 86T, 88T, 202T2 and 203T3.

**Table:** 

Ingredient	Company	Volume (μl)
Water	-	6.2
Phusion HF buffer (5x)	Thermo Fisher Scientific	4.0
dNTP-mix (10 μM each)	Thermo Fisher Scientific	1.6
Primer forward (10 μM)	Sigma-Aldrich	3.0
Primer reverse (10 μM)	Sigma-Aldrich	3.0
Template DNA	-	2.0
Phusion HF Polymerase	Thermo Fisher Scientific	0.2
Total	-	20.0

Step	Duration (s)	Cycles
Initial melting (98°C)	300	1
Melting (98°C)	30	35
Annealing (60°C)	30	
Elongation (72°C)	45	
Final elongation (72°C)	300	1
Final cooling (4°C)	-	1

Amplification products were purified with the QIAquick PCR purification kit (Qiagen), and pooled in roughly equimolar quantities. Pooled amplicon DNA was quantified (dsDNA BR assay, Thermo Fisher Scientific), purified, libraries prepared (NEBNext Sanger Sequencing Kit, New England Biolabs, Ipswich, MA, USA), and index tags applied (Sanger 168 tag set). Index tagged samples were amplified (8 cycles of PCR, KAPA HiFi kit, KAPA Biosystems), quantified by qPCR (KAPA Library Quant Kit, KAPA Biosystems) and submitted to cluster formation for MiSeq sequencing (300 BP PE read length, Illumina).

12,831,254 sequence reads were obtained and aligned to 2000 BP windows around each of the 96 SNV loci in the Devil7.1 reference using BWA-MEM (Li, 2013); the 95 loci (one PCR failed) had a median read coverage of 70,941 X (range 1,730 X to 481,111 X). We manually inspected each of the 95 loci using the Integrative Genomics Viewer (IGV) (Thorvaldsdóttir et al., 2013) to ensure alignment accuracy. As the template DNA used in this experiment was a pool of DNA from four tumors, and each SNV was predicted to be present, at least in the heterozygous state, in at least two of the four tumors, the minimum variant allele fraction (VAF), for the predicted alternative allele was expected to be 0.25. In order to distinguish true alleles from background sequencing errors, we first used alleleCount (https://github.com/cancerit/alleleCount) to calculate VAF for the two nucleotide bases that were neither the reference allele nor predicted alternative allele. We fitted a gamma distribution to these “background VAFs” and used this distribution to test if our predicted alternative allele VAF was significantly different to background. Predicted alternative alleles with VAF values that fell above 95% of the cumulative probability under the gamma curve were defined as validated SNVs. Overall, 93/95 SNVs were validated, detailed in Table S2.

#### SNV Genotyping across Normal Panel

We PCR screened each of the 93 validated SNVs across a panel of 30 normal devils to confirm genotyping accuracy. Whole genome amplified DNA from 30 devils was distributed with equal volume into three pools of 10 devils (Table S2). PCRs were performed, amplicons were pooled, libraries prepared and MiSeq sequencing performed (see section SNV Validation) with 300 BP PE reads. 12,116,462 sequence reads were generated, and mapped to 2000 BP windows around each of the 95 SNV loci in Devil7.1 using BWA-MEM (Li, 2013) with a median read depth of 80,778 X (range 1,594 X to 328,569 X). Using the same approach outlined above (see section SNV Validation), we obtained the classification results summarized in Table S2.

#### SNV Genotyping of *WWC3* and *MPDZ* Mutations

To assess the somatic pervasiveness of hemizygous *WWC3* SNV (DFT1) and *MPDZ* indel (DFT2) predicted loss-of-function mutations, we used the following sequencing approach. We PCR amplified (primers *WWC3* forward and *WWC3* reverse, see Key Resources Table) a 200 BP region around the affected *WWC3* locus on exon 21 in ten tumors 36T2, 85T, 86T, 87T, 88T, 95T, 96T, 221T, 331T and 333Ta which cover a wide spatiotemporal range (Table S2). Similarly, a region around the frameshift *MPDZ* indel on exon 9 was amplified (primers *MPDZ* forward and *MPDZ* reverse, see Key Resources Table) in the four DFT2 tumors 202T2, 203T3, 338T and 339T (Table S2). PCR products were cleaned up with the QIAquick PCR Purification kit (Qiagen). Products were then capillary sequenced with the corresponding PCR primers *WWC3* forward and *MPDZ* forward (Key Resources Table).

**Table:** 

Ingredient	Company	Volume (μl)
Water	-	5.5
PCR buffer (10x)	Qiagen	5.0
dNTP-mix (2.5 μM each)	Qiagen	4.0
Primer forward (10 μM)	Sigma-Aldrich	7.5
Primer reverse (10 μM)	Sigma-Aldrich	7.5
Template DNA	-	20.0
Taq Polymerase	Qiagen	0.5
Total	-	50.0

Step	Duration (s)	Cycles
Initial melting (94°C)	300	1
Melting (94°C)	30	35
Annealing (58°C)	30	
Elongation (72°C)	15	
Final elongation (72°C)	300	1
Final cooling (4°C)	-	1

#### SV Validation

Candidate Structural Variants (SVs) were validated with PCRs spanning breakpoints. PCR primers are listed in Table S5, and PCR conditions are listed below. Of the 345 candidate SVs, 345 (100%) were validated (Table S5). Candidate somatic SV amplicons were sequenced on the Illumina MiSeq platform. Amplicons were purified and libraries generated as described above in SNV Validation and were sequenced with 300 BP PE reads.

**Table:** 

Ingredient	Company	Volume (μl)
Water	-	8.3
CoralLoad buffer (10x)	Qiagen	2.0
dNTP-mix (10 μM each)	Thermo Fisher Scientific	1.6
Primer forward (10 μM)	Sigma-Aldrich	3.0
Primer reverse (10 μM)	Sigma-Aldrich	3.0
Template DNA	-	2.0
Taq Polymerase	Qiagen	0.1
Total	-	20.0

Step	Duration (s)	Cycles
Initial melting (94°C)	300	1
Melting (94°C)	30	35
Annealing (60°C)	30	
Elongation (72°C)	90	
Final elongation (72°C)	300	1
Final cooling (4°C)	-	1

#### SV Genotyping across Normal Panel

We screened all PCR validated SVs across a panel of 34 normal devil genomes. Briefly, whole genome amplified DNA from 34 devils was pooled in equal volume into four pools (3 pools of 10 devils, and 1 pool of 4 devils that comprised DNA from 202H1, 203H, 31H and 91H; Table S2) and PCRs were conducted with the reagents and conditions as described above (see section SV Validation). SVs that amplified in any one of the normal pools were classed as germline, and those which were not amplified in any of the normal pools were retained as candidate somatic. The SVs predicted to be unique to a single tumor were validated by confirming their absence by PCR in other tumors (Table S5).

#### SV Genotyping of *PDZD11*-*RFX2*

In order to establish the somatic pervasiveness of a detected intron-to-intron structural variant interlinking genes *PDZD11* and *RFX2* across the DFT1 tumor phylogeny, we used a similar PCR strategy as described above for the *WWC3* and *MPDZ* mutation screening. Briefly, a 231 BP amplicon involving breakpoints on chromosomes 2 and X, was obtained in eight additional DFT1 tumors 36T2, 85T, 87T, 95T, 96T, 221T, 331T and 333Ta (Table S2). Primers used were *PDZD11*-*RFX2* forward and *PDZD11*-*RFX2* reverse (Key Resources Table), and PCR conditions are listed below.

**Table:** 

Ingredient	Company	Volume (μl)
Water	-	5.3
CoralLoad buffer (10x)	Qiagen	2.0
dNTP-mix (10 μM each)	Thermo Fisher Scientific	1.6
Primer forward (10 μM)	Sigma-Aldrich	3.0
Primer reverse (10 μM)	Sigma-Aldrich	3.0
Template DNA	-	5.0
Taq Polymerase	Qiagen	0.1
Total	-	20.0

Step	Duration (s)	Cycles
Initial melting (94°C)	300	1
Melting (94°C)	30	35
Annealing (60°C)	30	
Elongation (72°C)	30	
Final elongation (72°C)	300	1
Final cooling (4°C)	-	1

#### DFT Diagnostic PCRs

A multiplex DFT diagnostic PCR has been developed to confirm DFT1 or DFT2 diagnosis (Kwon et al., 2018). Briefly, the PCR incorporates three primer sets, respectively targeting a DFT1-specific structural variant, a DFT2-specific structural variant, and the *RPL13A* locus, which acts as an internal positive control. The PCR was performed as described (Kwon et al., 2018).

#### Y Chromosome PCRs

Samples included in this analysis are listed in Table S6. Whole genome amplified DNA was used as a template for amplification of *SRY* or a set of DFT1/DFT2 diagnostic markers as follows with primers *SRY* forward and *SRY* reverse (Key Resource Table).

**Table:** 

Ingredient	Company	Volume (μl)
Water	-	11.3
PCR buffer (10x)	Qiagen	2.0
dNTP-mix (10 μM each)	Thermo Fisher Scientific	1.6
Primer forward (10 μM)	Sigma-Aldrich	1.5
Primer reverse (10 μM)	Sigma-Aldrich	1.5
Template DNA	-	2.0
Taq Polymerase	Qiagen	0.1
Total	-	20.0

Step	Duration (s)	Cycles
Initial melting (94°C)	300	1
Melting (94°C)	30	38
Annealing (64°C)	30	
Elongation (72°C)	90	
Final elongation (72°C)	300	1
Final cooling (4°C)	-	1

#### Drug Screen

##### Automated High-throughput Screen

Details of cell lines used in drug screen are presented in Table S7. Cells were seeded into 384-well plates using a XRD-384 (FluidX, Brooks Automation, Chelmsford, MA, USA) reagent dispenser. The number of cells seeded was individually optimized for each cell line to maximize the dynamic range of the assay: 85T = 600, 86T = 1200, 87T = 2000, 88T = 1600, 203T3 = 3200, 809T = 1600. Compounds were stored in Storage Pods (Roylan Developments, Fetcham, UK) providing a moisture-free, low oxygen environment, and protection from UV damage. Compounds were screened using a 7-point dose response curve and a linear half-log dilution series covering a 1000-fold concentration range. The dosing of the compounds was carried out using an Echo 555 (Labcyte, San Jose, CA, USA) acoustic dispenser and the duration of drug treatment was 144 hr (6 days). Cell number at the end of 6 days was measured using CellTitre-Glo 2.0 (Promega, Madison, WI, USA) reagent. 85T, 86T, 87T and 88T cell lines were screened as a single technical replicate in each of two separate screening runs. 203T3 cell line was screened in duplicate in each of two separate screening runs. 809T was screened in duplicate in each of two separate screening runs for a proportion of compounds, and was screened as a single replicate on the remaining proportion of compounds. Fluorescence intensity data from screening plates for each dose response curve was fitted using a multi-level fixed effect model (Vis et al., 2016).

##### Manual Follow-up Screen

We performed a follow-up drug screen to further elucidate the drug sensitivity of DFT1 cell lines to dual EGFR and ERBB2 inhibitors. Specifically, DFT1 showed particular sensitivity to Afatinib, an inhibitor of both ERBB2 and EGFR (Table S7). The observation that DFT1 cell lines were resistant to Gefitinib, an inhibitor of EGFR, suggests that the sensitivity to Afatinib is mediated by ERBB2. We further tested this hypothesis by manually screening DFT1 cell lines (85T, 86T, 87T, 88T) and three human cancer cell line controls (A549, AU565 and PC-9) with Erlotinib and Gefitinib (EGFR inhibitors), and Lapatinib and Afatinib (EGFR and ERBB2 inhibitors), as displayed in Table S7.

The control human cancer cell lines have the following known sensitivities. PC-9 has a drug sensitive deletion in EGFR (E746-A750 in exon 19) and is thus susceptible to EGFR inhibitors (Bean et al., 2007, Li et al., 2008). PC-9 cells are known to be highly sensitive to Gefitinib, Afatinib, and Erlotinib while exhibiting only a very modest sensitivity to Lapatinib (Bean et al., 2007). AU565 is an ERBB2-dependent breast cancer cell line and as such is sensitive to the ERBB2 inhibitors Afatinib and Lapatinib, but is insensitive to drugs targeting only EGFR. A549 is a human lung adenocarcinoma cell line with an oncogenic KRAS G12S point mutation, displaying resistance to both EGFR and ERBB2 inhibitors (Li et al., 2008). DFT1 cell lines and A549 were grown in DMEM/F-12 media, AU565 and PC-9 were grown in RPMI-1640 media. All cells were maintained at 37°C and 5% CO_2_.

Dose-response curves were obtained by setting up 96-well cell culture plates for each drug. DFT1 cells in each well were dosed with drugs at exponentially decreasing concentration. The maximum drug concentration was 1 μM. The cells were cultured in the presence of drugs for 48 hr. A fluorescence-based live-cell assay (Thermo Fisher Scientific) was used to detect cell viability. After live-cell detection, the cells were fixed overnight. On the next day, the cells were prepared for fixed cell detection. Cells were washed two times with 200 μl/well of water and stained with Syto60 1:5000 (Thermo Fisher Scientific). After 1 hr of incubation at room temperature, plates were washed two times with 200 μl/well of water. Lastly, 100 μl of water was added to each well and the plate was detected. The experiment was repeated in triplicates for each cell line and drug. IC_50_ values from this experiment are shown below as log_*e*_(μM) concentrations, indicate that DFT1 is resistant to Erlotinib, and that 3 of 4 DFT1 cell lines here tested show sensitivity to Lapatinib under these conditions (Table S7).

### Quantification and Statistical Analyses

Bioinformatics downstream analyses of ancestral data, mutational calls and drug screen results were performed in the R language for statistical computing (R Core Team, 2015), using existing Bioconductor libraries (Huber et al., 2015) and customized scripts.

#### Devil Population Analysis

We genotyped tumors 86T and 88T (DFT1), tumors 202T2 and 203T3 (DFT2), and normal devils 202H1, 203H and 91H against a panel of previously ascertained variants (Brüniche-Olsen et al., 2016). Of the 2,281 variants described by Brüniche-Olsen et al., we excluded (i) indels, (ii) single nucleotide polymorphisms (SNPs) falling into RADseq-fragments ambiguously mapping to the reference genome (i.e. >2 mismatching bases or one or more alignment gaps of total length >2 BP), (iii) SNPs mapping in windows of 5 BP around simple repeats, 500 BP around contig ends or 1,000 BP around scaffold ends, (iv) SNPs mapping to the X chromosome and (v) SNPs falling into regions which are non-diploid in any of the tumor samples (Table S4). In addition, using the genotypes provided by Brüniche-Olsen et al., we excluded those SNPs which showed limited variation across the population. Specifically, for each SNP we computed the proportion of individuals that shared identical genotypes. SNPs were ranked by the proportion of individuals sharing identical genotypes, and those SNPs which were within the group of 60% least varying across the population were excluded. Finally, if >1 SNP mapped to the same RADseq fragment, only the SNP mapping closest to the 5’ end of the fragment (with respect to the reference) was selected for further analysis. These steps provided a final set of 320 SNPs.

Of the 527 individuals genotyped in (Brüniche-Olsen et al., 2016), we excluded any individual with missing genotype data at more than 20% of loci. For the remaining 398 individuals, we extracted the genotypes assigned in (Brüniche-Olsen et al., 2016). Genotypes across DFT1 tumors 86T and 88T, DFT2 tumors 202T2 and 203T3, as well as normal devils 91H, 202H1 and 203H were genotyped at the 320 SNP loci using alleleCount (https://github.com/cancerit/alleleCount). Sites with <7 read coverage were marked as missing data, and remaining sites were coded as follows:

(i) homozygous 1/1: >70% reads support allele 1
(ii) heterozygous 1/2: >30% and <70% reads support alleles 1 and 2
(iii) homozygous 2/2: >70% reads support allele 2

Our 1 and 2 allele definitions were used as per Brüniche-Olsen et al. (2016). Missing genotypes across all 405 individuals were imputed by adopting the genotypes of the closest related SNP, as measured by Euclidian distance across the sample set.

Hierarchical clustering was then performed by applying the default R hclust() function (method: 'complete'), defining each genotype value as follows:

(i) homozygous 1/1: 0
(ii) heterozygous 1/2: 0.5
(iii) homozygous 2/2: 1

#### SNV and Indel Analysis

##### SNV and Indel Calling

We used Platypus version 0.8.1 for detecting and genotyping single nucleotide variants (SNVs) and small insertions and deletions (indels) (Rimmer et al., 2014). Variants were ascertained from the high-coverage genomes sequenced in this study (86T, 88T, 202T2, 203T3, 202H1, 203H) as well as from two previously sequenced devil genomes (31H, 91H) (Tables S1 and S2). Platypus was run twice on each BAM file with two different settings: (i) default mode with additional flags --minReads=3 and --minPosterior=0, (ii) default mode with --minReads=3, --minPosterior=0, --minFlank=0 and --trimReadFlank=10. Variants flagged with badReads, MQ, strandBias, SC and QD were removed, and remaining variants were merged into a single file and genotyped across each sample. Genotyped variants flagged with badReads, MQ, strandBias, SC and QD were removed for both SNVs and indels. The final variant list contained 1,882,666 SNVs and 356,570 indels genotyped across the set of tumors (86T, 88T, 202T2, 203T3) and hosts (202H1, 203H, 91H, 31H). The following post-processing steps were applied to our set of genotyped SNVs and indels.

(i) Homozygous-variant-in-reference filter. Sample 91H was used to assemble the Tasmanian devil reference genome (Murchison et al., 2012). This implies that variants called with a high variant allele fraction (VAF, proportion of reads at a base position supporting the variant allele) in this sample are likely to represent reference assembly errors. Thus, SNVs and indels called with VAF >0.9 in sample 91H were discarded from our variant list.
(ii) Strand bias filter. In regions with total coverage ≥11 across all eight samples, we rejected variant calls with less than 20% support on either forward or reverse sequencing strands. In regions with total coverage <11 reads across all samples, we removed variants that had less than two supporting reads in either forward or reverse direction.
(iii) Sequencing noise filter. Low-VAF SNVs and indels were found across all samples, including hosts, and therefore likely reflect consistent sequencing noise or alignment artefacts in these positions. A variant with VAF <0.2 in all samples with ≥1 supporting reads was discarded if at least one of the host samples had ≥1 supporting reads.
(iv) Simple repeats regions filter. SNVs and indels lying within a 5 BP window around simple repeat regions, as annotated by Tandem Repeat Finder (Benson, 1999), were discarded.
(v) Regions filter. The Tasmanian devil reference genome (Devil7.1) is a scaffold-level assembly, consisting of 237,291 contigs assembled into 35,974 scaffolds. We rejected any variant mapping within 500 BP from the start or end of a contig, or within 1000 BP from the start of end of a scaffold. In addition, variant calls mapping to scaffolds not assigned to a chromosome were discarded.

Combined, these filtering steps left 988,972 SNVs and 194,250 indels. We further genotyped these across our panel of 30 low-coverage normal devil genomes and 12 previously published normal devil genomes (Wright et al., 2017) using Platypus with settings --minPosterior=0 and --minReads=0.

##### SNV and Indel Subsetting

We classified our variants into different categories, outlined below. Number of variants in each set is indicated in table below.

(i) Germline variants, which are present in the Tasmanian devil population, were defined as variants which had ≥5 supporting sequence reads in high-coverage normal genomes 91H, 202H1 or 203H, or ≥1 supporting sequence read in genome 31H and 42 low-coverage normal genomes (samples listed in Table S2).
(ii) Potentially somatic variants are shared between both tumors of the same lineage, or all four tumors, with ≥5 reads in each tumor, but have <5 reads in each of the three high-coverage normal devil genomes 91H, 202H1 and 203H, and 0 reads in genome 31H and 42 low-coverage normal devil genomes (Table S2). This set includes the following three subsets:

###### DFT1 Potentially Somatic Variants

Present with ≥5 reads in 86T and 88T, but ≤5 reads in DFT2 tumors and 202H1, 203H and 91H normal devil genomes, and 0 reads in genome 31H and 42 low-coverage normal devil genomes (Table S2). These represent both germline variation that was inherited by the DFT1 founder devil, but that is not captured in the DFT2 founder devil, nor in the normal genomes examined here; and somatic variants that were acquired before divergence of 86T and 88T.

###### DFT2 Potentially Somatic Variants

Present with ≥5 reads in 202T2 and 203T3, but ≤5 reads in DFT1 tumors and normal devil genomes 202H1, 203H and 91H, and 0 reads in genome 31H and 42 low-coverage normal devil genomes (Table S2). These represent both germline variation that was inherited by the DFT2 founder devil, but that is not captured in the DFT1 founder devil, nor the normal genomes examined here; and somatic variants that were acquired before divergence of 202T2 and 203T3.

###### DFT1 and DFT2 Potentially Somatic Variants

Present with ≥5 reads in 86T, 88T, 202T2 and 203T3, but ≤5 reads in normal devil genomes 202H1, 203H and 91H, and 0 reads in genome 31H and 42 low-coverage normal devil genomes (Table S2). These potentially represent both germline variation that was inherited by both the DFT1 and DFT2 founder devil, but that is not captured in the normal genomes examined here; and somatic variants that were acquired by DFT1 before the divergence of 86T and 88T, and were also independently acquired by DFT2 before the divergence of 202T2 and 203T3.

(iii) Tumor-unique variants are those variants that are present with ≥5 reads in only one tumor, and are supported by <5 reads in every other tumor and normal genomes 91H, 202H1 and 203H, as well as 0 reads in genome 31H and 42 additional normal genomes (Table S2). These variants could be newly arising somatic mutations that occurred after divergence of 86T-88T or 202T2-203T3 from their most recent common ancestor (MRCA) tumors; or germline variants inherited by the DFT1 or DFT2 founder devils but not shared with the normal panel, or somatic mutations that arose before the MRCA of 86T-88T or 202T2-203T3, that were subsequently lost in one tumor due to back mutation or copy number loss.
(iv) Remainder variants comprise SNVs or indels which are either (i) represented by support from ≥5 reads in at least one DFT1 and one DFT2 tumor, but not found with ≥5 reads in all four tumors, not found with ≥5 reads in any high-coverage normal genomes (202H1, 203H, 91H) or with >0 reads in 31H and 42 low-coverage hosts (Table S2); or (ii) supported by <5 reads in ascertainment panel samples (86T, 88T, 202T2, 203T3, 202H1, 203H, 91H).

The table below indicates number of variants belonging to each category outlined above. Panels on right indicate with “x” the presence and "-" the absence of variants belonging to each category within each sample.

**Table:** 

Set	SNVs	Indels	86T	88T	202T2	203T3	Normal Devils
Total	988,972	194,250					
Germline	974,040	191,001	x	x	x	x	x
DFT1 potentially somatic DFT2 potentially somatic DFT1/DFT2 potentially somatic	2,796 3,503 88	387 549 23	x - x	x - x	- x x	- x x	- - -
86T tumor-unique 88T tumor-unique 202T2 tumor-unique 203T3 tumor-unique	3229 3583 231 398	477 566 19 66	x - - -	- x - -	- - x -	- - - x	- - - -
Remainder	1,104	1,162	x	x	x	x	x

Genome browser visual assessments of 75 individual variant calls yielded false positive call rates of <5% for SNVs (2/75) and <15% for indels (9/75).

##### SNV and Indel Annotation

Of the 602 genes in the COSMIC Cancer Gene Census (http://cancer.sanger.ac.uk/census/; downloaded on 17/05/2016), 490 were annotated in the Ensembl Devil7.0 genebuild (http://www.ensembl.org/Sarcophilus_harrisii/Info/Index). An additional 69 genes were annotated only in the NCBI 101 annotation gene set (https://www.ncbi.nlm.nih.gov/genome/annotation_euk/Sarcophilus_harrisii/101/; downloaded on 17/05/2016), but were not in the Ensembl gene set; thus, 43 cancer genes were not detectable in the Tasmanian devil reference genome (Table S3). SNV and indel subsets were annotated with the Ensembl variant effect predictor (VEP) using default settings (McLaren et al., 2010) (Tables S2, S3, and S4). We also ran an alternative variant caller, SAMtools mpileup, specifically on the COSMIC Cancer Gene Census gene set (Table S3) and searched manually for additional protein-altering variants, however, this did not detect additional variants.

##### SNV-Based Tumor Purity Estimation

Tumor DNA sequenced in this study was derived from cell lines, and thus is likely to be relatively pure. However, it is possible, particularly for early passage cell lines, that host cells remain in culture. We assessed the purity of 86T, 88T, 202T2 and 203T3 by examining VAF of germline SNVs. This analysis revealed that 86T, 88T and 202T2 contain only tumor DNA, whereas 203T3 had approximately 5-10% host DNA at the time when DNA was collected for sequencing.

#### Copy Number Analysis

##### Scaffold Exclusion

The Tasmanian devil genome Devil7.1 has 35,974 scaffolds, most of which are assigned to chromosomes (Murchison et al., 2012), with scaffolds ordered along chromosomes using synteny with the opossum genome (Mikkelsen et al., 2007, Murchison et al., 2012). Short scaffolds, for which synteny with the opossum genome could not be determined, are placed at the end of each chromosome. We excluded these latter scaffolds from copy number analysis, together with the entire X chromosome. Coordinates of excluded scaffolds are listed below.

**Table:** 

Chromosome	Excluded Scaffolds
1	Chr1_supercontig_000000399 to Chr1_supercontig_000006728
2	Chr2_supercontig_000000501 to Chr2_supercontig_000008380
3	Chr3_supercontig_000000417 to Chr3_supercontig_000007196
4	Chr4_supercontig_000000317 to Chr4_supercontig_000006728
5	Chr5_supercontig_000000218 to Chr5_supercontig_000003187
6	Chr6_supercontig_000000194 to Chr6_supercontig_000002843
X	Chrx_supercontig_000000000 to Chrx_supercontig_000002377
Un	ChrU_supercontig_000000000 to ChrU_supercontig_000000439

##### Copy Number Calling

We used the read-depth based algorithm cn.MOPS to assign copy numbers to genomic segments of our four high-coverage tumor genomes (Klambauer et al., 2012). Samples 91H, 202H1 and 203H served as normal controls. Briefly, read-depths were counted in 500 BP bins across selected scaffolds using cn.MOPS getReadCountsFromBAM(), and coverage was normalized to the mode. After modelling copy number posterior likelihoods between copy number (CN) 0 and CN6 for each 500 BP bin in each sample, the cn.MOPS circular binary segmentation algorithm was invoked with a 3 x 500 BP minimum length parameter for non-CN2 segments.

##### Copy Number Filtering

Each candidate copy number variant (CNV) (defined as a segment with CN≠2) was filtered through a number of steps. First, the minimum size of copy number changes specific to a unique tumor within either the DFT1 or DFT2 lineage (tumor-unique CNVs) was set to 5000 BP, i.e. at least 10 neighboring bins of 500 BP. To further validate tumor-unique CNV segments, we conducted quantitative, lineage-specific sequence read count comparisons. CNVs were only retained when their dispersions significantly differed (p<0.01) between 86T-88T or 202T2-203T3, as measured by a paired two-sided Student's t-test. For segments with insignificantly differing read count distributions, copy number posterior likelihoods from cn.MOPS were pooled between both tumors. The highest scoring median value was then chosen for assigning the same segmental copy number to both 86T and 88T or 202T2 and 203T3. Table S4 lists copy number segments and assignments.

##### Copy Number Annotation

Non-diploid copy number segments were intersected with the set of Ensembl genes (Devil7.0) (Tables S3 and S4). Genes that were completely or partially represented on non-diploid segments, such that loss of one copy or gain of one or more copies was predicted, were considered to be involved in a CNV (Figure 4A, Tables S3 and S4).

To validate gene copy number annotations in COSMIC Cancer Gene Census genes (http://cancer.sanger.ac.uk/census/; downloaded on 17/05/2016), and to obtain calls of those COSMIC genes falling into previously excluded scaffolds (see Scaffold Exclusion), and which are not annotated by Ensembl, we conducted an independent, parallel copy number assessment (Table S3). 559/602 COSMIC Cancer Gene Census genes are annotated in the devil reference genome in the Ensembl and/or NCBI gene sets (see SNV and Indel Annotation). To search for the remaining 43 genes, which were annotated neither by Ensembl nor NCBI, we obtained transcript sequences for each gene’s opossum – or if this was not available – human orthologue. We used BLAT (Kent, 2002) to align the gene transcript to the devil genome; this approach allowed us to preliminary annotate an additional 4 genes. Next, each gene’s footprint was defined as the genomic interval between the start of the first exon and the end of the last exon of each gene. Gene footprints were divided into bins of 500 BP, or – in the case when the gene region would be partitioned into fewer than 10 bins – into bins of 50 BP. For each bin in each sample, the average coverage was collected from the aligned reads using the SAMtools bedcov function (Li et al., 2009). Samples were divided into the following groups: DFT1 (86T, 88T), DFT2 (202T2, 203T3) and host (31H, 91H, 202H1, 203H). An ANOVA test was used to identify gene loci with a heterogeneous distribution of coverage, where the mean of one group differed significantly from the other two with a confidence level of 0.0001. Tukey's range test was then performed to establish which samples had a different mean. A threshold difference of 0.25 was used in order to call a copy number gain or loss after a significant difference was determined. This threshold was also used to assign individual copy number variants to specific samples.

##### CNV Genotyping across Normal Panel

We analyzed copy number changes on chromosome 3 in our panel of 46 normal devil genomes as follows. Sequencing reads falling into 10,000 BP windows tiled along the chromosome were counted by cn.MOPS getReadCountsFromBAM() (Klambauer et al., 2012). Bin counts were normalized by the average sequencing depth across the whole respective sample, as listed in Table S2.

#### Structural Variant Analysis

##### Structural Variant Calling

We used Breakpoints via Assembly (BRASS), a tool that uses discordantly mapped read pairs, for detecting structural variants (SVs). A minimum of two discordant reads detecting a breakpoint in any one sample was required to make a call. SVs were ascertained from tumors 86T, 88T, 202T2 and 203T3, and normal genomes 91H, 31H, 202H1 and 203H.

##### Structural Variant Filtering

We rejected SV calls for which at least one end fell within a scaffold not assigned to a chromosome. Only calls with a total of >10 supporting reads across all eight samples (86T, 88T, 202T2, 203T3, 31H, 91H, 202H1, 203H) were retained. Moreover, any SV prediction with >2 combined supporting reads across any of the four normals 31H, 91H, 202H1, and 203H was discarded as a likely germline polymorphism. Somatic and potentially somatic SVs were defined as having >10 supporting reads in individual tumors or both tumors of a lineage respectively, together with <3 supporting reads in all other samples combined.

##### Structural Variant Display

Circos plots of the set of SVs that were not detected in the normal panel are displayed in Figure 3B using the R circlize package (Gu et al., 2014).

##### Structural Variant Breakpoint Assembly

Exact breakpoint types and corresponding single-base resolution were reconstructed through an in-house analysis pipeline centered around the TIGRA assembler (Chen et al., 2014). Briefly, the structural variant breakpoint predictions identified by BRASS were given as input to TIGRA. TIGRA was used to select structural variant-supporting reads from tumors (86T, 88T, 202T2 and 203T3), and from them assemble contigs spanning the structural variant breakpoints. These contigs were realigned to the devil reference sequence using BWA-MEM (Li, 2013). We selected those contigs that mapped to both scaffold locations predicted by BRASS. We analyzed these alignments to determine the precise location of the breakpoint, to base pair resolution, and to categorize each as either non-templated sequence insertions, microhomologies, or blunt-end breakpoints (Table S5). Of these selected contigs, those with the highest scoring alignments were aligned against the MiSeq amplicon reads. The resulting contig-amplicon read alignments were manually inspected using IGV to further validate the breakpoint junction sequences (Thorvaldsdóttir et al., 2013). As an additional check, the results obtained through our TIGRA pipeline were also reproduced using the assembly based structural variant caller SvABA (Wala et al., 2018).

##### Structural Variant Annotation

SV breakpoints were intersected with Ensembl gene predictions. SVs that were predicted within a gene footprint are annotated in Table S5. Strand and frame information was used to predict the potential for SVs to create in-frame fusion genes (Tables S3 and S5).

#### Mutational Signature Analysis

##### SNV Spectra for Somatic Mutational Signatures

The set of tumor-unique SNVs for each tumor (see SNV and Indel Subsetting) were extracted, together with their immediate 5’ and 3’ contexts (96 mutation types). 86T and 88T tumor-unique variants were pooled, and 202T2 and 203T3 tumor-unique variants were pooled, generating DFT1 and DFT2 somatic mutation sets, respectively. Triplet frequency normalization was done as follows. We counted frequencies of the 32 pyrimidine-context nucleotide triplet combinations in the variant-calling accessible (see SNV and Indel Calling) Devil7.1 reference. Each of the 96 observed mutation counts were then divided by its corresponding triplet frequency, prior to rescaling the sum of mutational proportions to 100%.

##### Normalization of COSMIC Mutational Signatures

The thirty consensus mutational signatures derived from human cancers which are available in the COSMIC database (http://cancer.sanger.ac.uk/cosmic/signatures; downloaded on 01/06/2017) and are relative to the human genome were normalized as follows: we counted frequencies of the 32 pyrimidine-context nucleotide triplet combinations in the human reference genome GRCh37 (hg19). Each of the 96 mutation proportions of each COSMIC signature were then divided by its corresponding triplet frequency, yielding a species-agnostic mutational signature, prior to rescaling the sum of mutational proportions to 100%.

##### Fitting COSMIC Mutational Signatures DFT1 and DFT2 Spectra

We developed a Bayesian multinomial mixture model to refit known COSMIC mutational signatures to devil DFT1 and DFT2 somatic spectra. The fitting is done using Markov Chain Monte Carlo sampling (MCMC), using the No-U-Turn sampler implemented in the Stan programming language (Carpenter et al., 2017). In the model, the mutational signatures are interpreted as the probability parameters of independent multinomial distributions, and the observed mutation counts in the 96 mutational categories are treated as draws from a mixture of these multinomials. The MCMC process samples mixture weights that specify the degree to which each signature contributes to the observed mutations. We use a symmetrical, uniform Dirichlet distribution as our prior on the mixture weights.

Model specification:

**Table:** 

W ∼ Dirichlet (1)	Prior on mixture weights
θ = WS	Multinomial mixture probabilities
M ∼ Multinomial (θ)	Likelihood

M: 1×96 vector of mutation counts by category;

1: 1×K vector, each entry is 1;

W: 1×K vector of mixture weights;

S: K×96 matrix of mutational signatures;

K: number of mutational signatures;

θ: 1×96 vector of multinomial probabilities resulting from the mixture of mutational signatures, S, according to weights, W.

Given that human signatures 1 and 5 are almost universal in human cancer and normal tissues (Alexandrov et al., 2013, Alexandrov et al., 2015a, Blokzijl et al., 2016, Ju et al., 2017, Rahbari et al., 2016), we first fitted human signatures 1 and 5 to pooled DFT1 (6,812 variants) and DFT2 (629 variants) variants.

Next, we assessed the improvement of fit when introducing the remaining 28 known human signatures. We assessed cosine similarities between the DFT-unique spectra and double-fits of signatures 1, 5, as well as of any triple-fits of signatures 1, 5, N ∈ [2-4,6-30] (Table S2). In order to avoid overfitting, we set a minimum threshold of 0.02 cosine similarity increase between 1, 5 and any 1, 5, N signature combinations for significance, as previously described (Schulze et al., 2015). However, only signature combinations 1, 5, 6 and 1, 5, 14 and 1, 5, 15 withstood this criterion in case of the fitting to the DFT1-unique spectrum (Δ0.0479 for signature combination 1, 5, 6; Δ0.0492 for signature combination 1, 5, 14; Δ0.0669 for signature combination 1, 5, 15), whereas no combinations surpassed Δ0.02 in the case of DFT2-unique variants (Table S2). As we did not detect the additional hallmarks of signature 6 and 15 (large numbers of small (<3 BP) indels at mono/polynucleotide repeats) or signature 14 (high numbers of somatic mutations (>200 per megabase), see http://cancer.sanger.ac.uk/cosmic/signatures; last access on 05/10/2017), we believe that it is unlikely that these signatures are present.

#### Virus Screen and PAV Analysis

##### De Novo Genome Assembly

*De novo* assemblies were produced from four tumor genomes (86T and 88T (DFT1) and 202T2 and 203T3 (DFT2)) and two host genomes (202H1 and 203H). We used Fermi (Li, 2012) to perform base error corrections for raw reads, to remove erroneous sequencing data and to generate a contig-wise assembly. We also ran Phusion2 (Mullikin and Ning, 2003) to obtain a second assembly with the base error corrected short reads. SOAPdenovo (Li et al., 2010) was used to process the cleaned reads in a third assembly run, which was improved using SSPACE (Boetzer et al., 2011). Next, Fermi/Phusion2 contigs were aligned to the SOAP scaffolds and assembly gaps closed when a piece of Fermi/Phusion2 sequence bridged two neighboring SOAP scaffolds.

##### Presence/Absence Variation (PAV) Analysis

Presence/absence variations (PAVs) are the sequences that are present in one genome assembly, but which are undetectable in another. We focused on identifying PAV contigs that were present one or more of the four tumor *de novo* assemblies, but which were absent from the reference genome. We first built an alignment index for absence in the reference assembly Devil7.1 using SMALT (https://sourceforge.net/projects/smalt/). In order to reduce CPU time, we shredded each tumor assembly into 1 kilobase fragments while removing ‘N’ bases, prior to alignment against the indexed absence (Devil7.1) assembly. Last, we filtered out small repetitive elements placed at ambiguous locations. We have integrated this software into a pipeline, scanPAV, which can be downloaded from https://github.com/wtsi-hpag/scanPAV/ (Giordano et al., 2018). This method produced a set of PAV candidate contigs which had evidence for presence in one or more tumor genome assembly, but which appeared to be absent from the devil reference genome. We further filtered these candidate tumor-specific PAV contigs by aligning sequence reads derived from the reference genome (91H) to them. Contigs with 91H sequence coverage >10 X were removed.

To further filter candidate PAV contigs for absence across a panel of normal devil genomes, the set of candidate tumor-unique PAV contigs were concatenated with Devil7.1 to create four Devil7.1+PAV assemblies, with each assembly carrying the set of PAVs unique to one of the four tumors. Next, we extracted the set of sequence reads from tumors 86T, 88T, 202T2 and 203T3 and normal devils 31H, 91H, 202H1 and 203H which previously did not map to Devil7.1, and aligned these to Devil7.1+PAV using BWA-MEM (Li, 2013). We measured the read depth of each candidate PAV contig in each sample, and retained those contigs that had read depth of at least 40% mean whole genome read depth in at least one tumor (thresholds were as follows, 86T – 34.4 X, 88T – 26.8 X, 202T2 – 26.8 X, 203T3 – 28.0 X), but that did not reach 20% whole genome read depth in any host (thus plausibly representing a single copy integration event in tumors but not in normal genomes); the thresholds for hosts were as follows: 31H – 3.4 X, 91H – 13.0 X, 202H1 – 9.8 X, 203H – 9.0 X. After this filtering, a total of 139 candidate tumor-specific PAV contigs remained (Table S1). The tumor-specificity of these contigs was assessed by aligning reads from the other three tumors to each individual tumor’s set of candidate PAVs. The contigs were further evaluated by comparing against the NCBI 'nt' sequence database with the default 'dc-megablast' option in BLAST+ 2.6.0 (Camacho et al., 2009). The top hit was annotated, including target species name, ID, BLAST identity, hit length, E-value and bitscore (Table S1).

##### Y Chromosome Contig Identification

We used genome assemblies of a male host, 202H1, as well as DFT2 tumors 202T2 and 203T3 to identify Y chromosome contigs that were present in these assemblies but which were absent in the female Tasmanian devil reference genome Devil7.1. Contigs identified in 202H1, 202T2 and 203T3 which were absent in the reference genome were screened using BLAT (Kent, 2002) for the presence of a ∼ 825 BP dasyurid-specific intron located within the *SRY* gene (O’Neill et al., 1998). As an input query, we used the intronic *SRY* sequence of the stripe-faced dunnart (*Sminthopsis macroura*). Identified sequences were used as seeds for alignments of the neighboring exons with *SRY* cDNA sequences identified in rock wallabies (O'Neill et al., 1997).

Contigs devil-202H_4481 (202H1, length: 84,660 BP), devil-202T_3709 (202T2, length: 84,684 BP) and devil-203T_28242 (203T3, length: 70,068 BP) were identified as Y chromosomal sequences harboring *SRY*.

#### Drug Screen IC_50_ Analysis

IC_50_ drug sensitivity values for different cell lines, as derived from our high-throughput screen, were used as an input for log_*e*_(IC_50_) hierarchical clustering. This was performed by applying the default R hclust() function (method: 'complete') on the Euclidian distance matrix derived from each pairwise drug and cell line combination. Figure 5B shows data for 6 DFT cell lines clustered with 104 compounds. IC_50_ data from human cell lines was obtained from the Genomics of Drug Sensitivity in Cancer (GDSC) database (http://www.cancerrxgene.org/, downloaded on 07/05/2017, Yang et al. (2013)).

### Data and Software Availability

The accession number for genomic data reported in this paper is ENA: PRJEB21902. Additional materials such as IHC and FISH images, mutational calls, Devil7.0 to Devil7.1 translations, genome assembly contigs and PAVs can be found on Mendeley Data (https://doi.org/10.17632/znfphvhmbv.1). Code used in this study is made available on Github (https://github.com/MaximilianStammnitz).

## Supplemental Information

Document S1. Figures S1 and S2

Table S1, Related to Figure 1

Table S2, Related to Figure 2

Table S3, Related to Figures 2, 3, and 4

Table S4, Related to Figures 2 and 4

Table S5, Related to Figures 3 and 4

Table S6, Related to Figure 4

Table S7, Related to Figure 5

Document S2. Article plus Supplemental Information
