# Supplementary material for: The Origins and Vulnerabilities of Two Transmissible Cancers in Tasmanian Devils
Source: Cancer Cell. 2018 Apr 9;33(4):607–619.e15. doi: 10.1016/j.ccell.2018.03.013 (PMC5896245; doi:10.1016/j.ccell.2018.03.013)
Supplement: Document S1. Figures S1 and S2 [file mmc1.pdf]

Cancer Cell, Volume *33*

Supplemental Information

The Origins and Vulnerabilities of Two Transmissible Cancers in Tasmanian Devils

Maximilian R. Stammnitz, Tim H.H. Coorens, Kevin C. Gori, Dane Hayes, Beiyuan Fu, Jinhong Wang, Daniel E. Martin-Herranz, Ludmil B. Alexandrov, Adrian Baez-Ortega, Syd Barthorpe, Alexandra Beck, Francesca Giordano, Graeme W. Knowles, Young Mi Kwon, George Hall, Stacey Price, Ruth J. Pye, Jose M.C. Tubio, Hannah V.T. Siddle, Sukhwinder Singh Sohal, Gregory M. Woods, Ultan McDermott, Fengtang Yang, Mathew J. Garnett, Zemin Ning, and Elizabeth P. Murchison

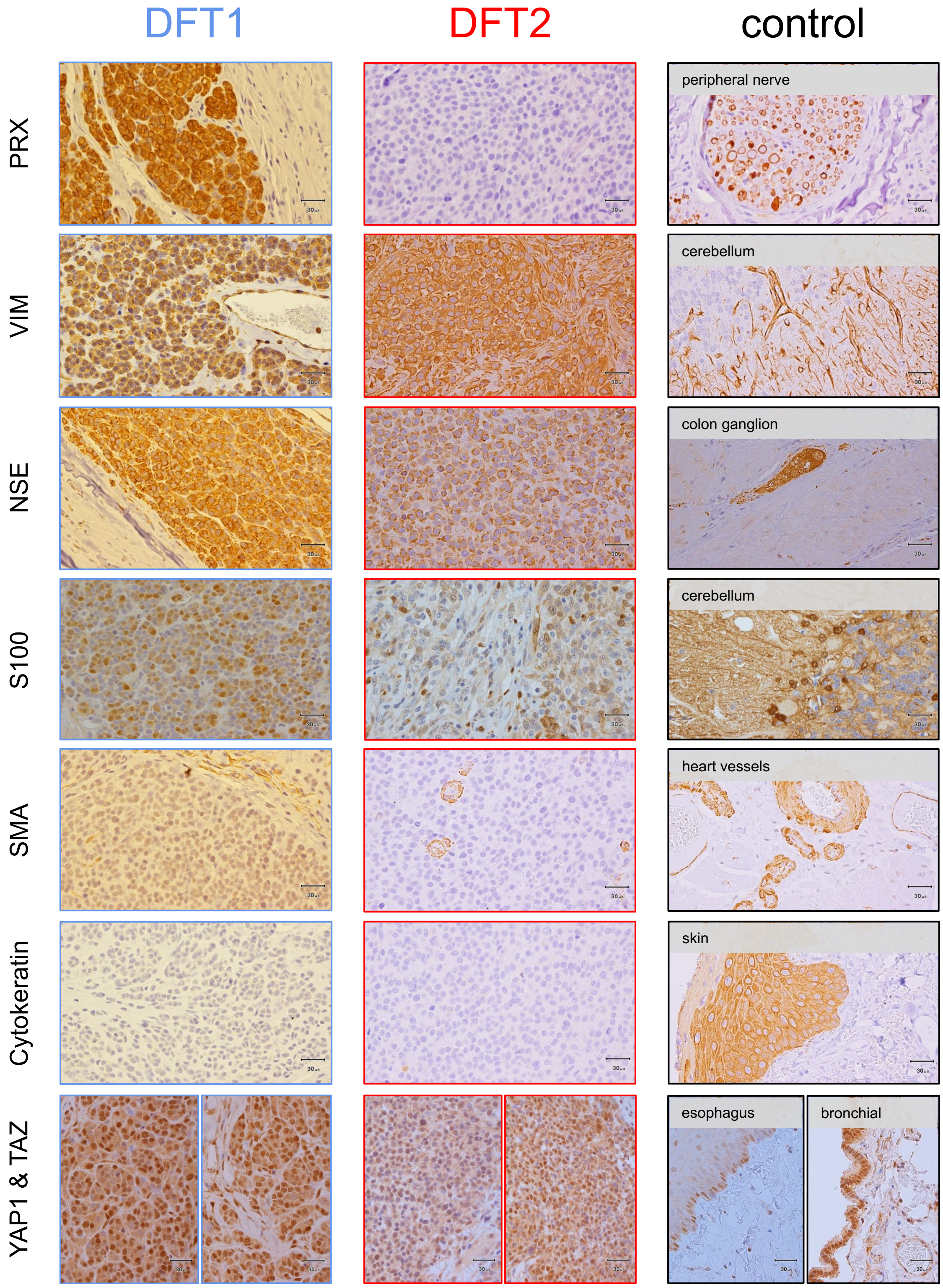

Figure S1, related to Figure 1: Immunohistochemistry.

DFT1 (left), DFT2 (center) and control (right) tissues stained with tissue lineage markers (Loh et al., 2006). PRX is included as a control (Pye et al., 2016b). Scale bar, 30 µm. PRX, periaxin; VIM, vimentin; NSE, neural specific enolase; SMA, smooth muscle actin; YAP1 (bottom left panels), yes-associated protein 1; TAZ/WWTR1 (bottom right panels), transcriptional coactivator with PDZ-binding motif/WW domain-containing transcription regulator 1. Nuclear localization of YAP1 and TAZ/WWTR1 indicates dephosphorylated, active states of the proteins (Moroishi et al., 2015; Wu et al., 2018).

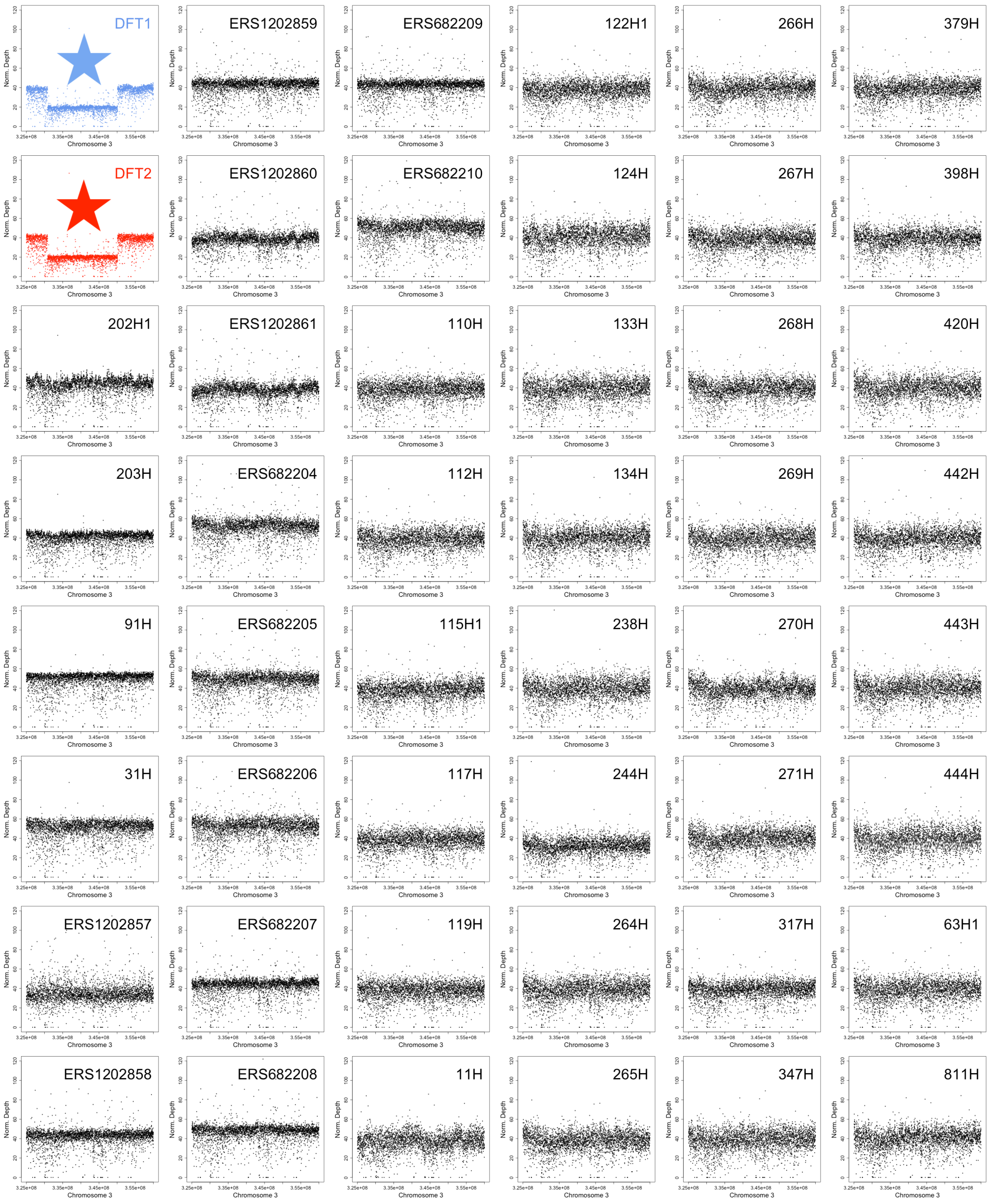

Figure S2, related to Figure 4: Genomic copy number at chromosome 3 locus in DFT1, DFT2 and 46 normal Tasmanian devil genomes.

Copy numbers of DFT1, DFT2 and 46 normal Tasmanian devil genomes at the chromosome 3 locus that has undergone hemizygous deletion in both DFT1 and DFT2 (Chr3_SC_000000273 position 360,001 to Chr3_SC_000000312 position 30,000). Dots represent number of sequence reads mapping in 10,000 base pair non-overlapping bins normalized to average sequencing depth. The region that has undergone hemizygous deletion in DFT1 and DFT2 is marked with a blue and red star, respectively.
